# A Global Methane Observation System to Reduce Uncertainty for Anthropogenic and Natural Sources and Sinks for Detecting and Attributing Climate Feedbacks

**DOI:** 10.1002/advs.76624

**Published:** 2026-07-29

**Authors:** P. Ciais, S. Peng, J. Chang, F. Li, Q. Zhu, K. Yuan, G. Hugelius, H. Li, Y. Cai, F. Chevallier, K. Tibrewal, E. A. Kort, K. Arndt, J. Watts, B. Buma, N. Besic, P. I. Palmer, H. Cadillo‐Quiroz, E. Euskirchen, M. J. Gondwe, A. Hoyt, R. Jackson, S. Malone, D. Monteverde, S. Natali, M. Ramonet, C. Rey‐Sanchez, L. B. Sagang, E. A. G. Schuur, R. Vargas, R. Varner, Z. Zhang, B. Poulter

**Affiliations:** ^1^ CEA CNRS UVSQ Centre Orme des Merisiers Laboratoire Des Sciences Du Climat et De L'environnement Gif sur Yvette France; ^2^ Sino‐French Institute For Earth System Science College of Urban and Environmental Sciences and Laboratory for Earth Surface Processes Peking University Beijing China; ^3^ College of Environmental and Resource Sciences Zhejiang University Hangzhou China; ^4^ Doerr School of Sustainability Department of Earth System Science Stanford University Stanford California USA; ^5^ Climate and Ecosystem Sciences Division Climate Sciences Department Lawrence Berkeley National Laboratory Berkeley California USA; ^6^ Department of Earth and Atmospheric Sciences University of Houston Houston Texas USA; ^7^ Department of Physical Geography Bolin Centre for Climate Research Stockholm University Stockholm Sweden; ^8^ Stockholm University Stockholm Sweden; ^9^ University of Michigan Ann Arbor Michigan USA; ^10^ Woodwell Climate Research Center Falmouth Massachusetts USA; ^11^ Environmental Defense Fund Boulder Colorado USA; ^12^ IGN ENSG Laboratoire D'inventaire Forestier (LIF) Nancy France; ^13^ National Centre For Earth Observation University of Edinburgh Edinburgh UK; ^14^ School of Life Sciences Arizona State University Tempe Arizona USA; ^15^ Biodesign Institute Arizona State University Tempe Arizona USA; ^16^ Institute of Arctic Biology University of Alaska Fairbanks Fairbanks Alaska USA; ^17^ Okavango Research Institute University of Botswana Maun Botswana; ^18^ Yale School of the Environment Yale University New Haven Connecticut USA; ^19^ Spark Climate Solutions San Francisco California USA; ^20^ Department of Marine, Earth, and Atmospheric Sciences North Carolina State University Raleigh North Carolina USA; ^21^ Institute of the Environment and Sustainability University of California Los Angeles California USA; ^22^ Ctrees Pasadena California USA; ^23^ Center For Ecosystem Science and Society Northern Arizona University Flagstaff Arizona USA; ^24^ Department of Earth Sciences and Institute for the Study of Earth, Oceans and Space University of New Hampshire Durham New Hampshire USA; ^25^ Department of Geological Sciences and Bolin Centre For Climate Research Stockholm University Stockholm Sweden; ^26^ National Tibetan Plateau Data Center State Key Laboratory of Tibetan Plateau Earth System, Environment and Resources (TPESER) Institute of Tibetan Plateau Research Chinese Academy of Sciences Beijing China

**Keywords:** atmospheric methane, atmospheric sciences, environmental science, flux, global warming, methane, satellite, tropics, warning system, wetland

## Abstract

Atmospheric methane (CH_4_) concentrations are accelerating global warming as net emissions increase. Observing systems that quantify sources remain too sparse and fragmented to detect trends—especially in remote regions where climate‐driven natural emissions may be rising. We provide a framework for quantifying uncertainty reductions through the implementation of a global ecosystem‐methane observing system designed to: (i) substantially lower uncertainty in sectoral and regional emissions, (ii) separate co‐occurring anthropogenic and natural fluxes, and (iii) trend detection at regional scales to verify mitigation progress and provide early warning of natural feedbacks. Using bottom‐up inventories and process‐model ensembles for 2014–2023, we show that anthropogenic emissions remain uncertain by ∼32% globally, while natural sources—tropical and boreal‐arctic wetlands, fires, and inland waters—carry far larger uncertainties (+ 70%) and trend uncertainties reaching ∼200%. Additional observations must match spatial emission structure to increase observability of emissions: high‐resolution satellite constellations for point sources combined with expanded flux networks and wetland mapping for diffuse sources, and denser ground‐based atmospheric column measurements to restore observability in under‐sampled tropics and high latitudes. Notional analyses indicate that targeted additions of flux towers and ∼20 in situ atmospheric column concentration instruments per key tropical region could reduce continental‐scale uncertainties at modest cost.

## Graphical abstract

Conceptual illustration of a Global Ecosystem Methane Observing System (GEM‐OS) integrating satellites, aircraft, atmospheric networks, and ecosystem measurements to quantify methane emissions from anthropogenic and natural sources. The multi‐scale observing framework improves source attribution, reduces uncertainty in regional methane budgets, and enables early detection of climate‐driven feedbacks from wetlands, fires, permafrost, agriculture, and fossil‐fuel emissions.

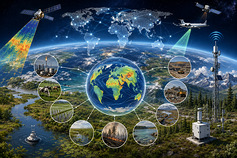

## 1 Introduction

Atmospheric methane (CH_4_) is the second largest contributor to anthropogenic global warming, with a global warming potential 80 times higher than carbon dioxide over a 20‐year horizon [1, 2]. Methane is accumulating in the atmosphere because emissions continue to exceed removals by oxidation from hydroxyl radicals (OH), chlorine radicals, and soil uptake, resulting in an atmospheric lifetime of ∼12 years. Rising methane emissions are driven by direct human activities from fossil fuel extraction and processing, livestock production, rice cultivation, aquaculture, anoxic decomposition of waste and wastewater, and by climate change‐driven increases of natural emissions including fires, wetlands and inland waters plus small sources such as termites, geological emissions and wild ruminants [3].

The Global Methane Pledge, endorsed in 2021 by 159 countries and the European Commission (https://www.globalmethanepledge.org), targets the reduction of those direct human‐induced emissions, while emissions from ‘unmanaged’ natural wetlands, rivers and fires are considered by countries to be outside their scope of action. However, the increase of methane after 2006, its acceleration in 2015, and further in 2020 is partly attributable to rising natural emissions [4, 5]. Models predict that climate‐driven emissions from wetlands and boreal wildfires will increase in the future, amplifying future warming, but large uncertainties remain on regional trends scales [6, 7, 8, 9]. In boreal forests, arctic tundra, and tropical forests, emissions from fires are expected to increase from more frequent and extreme hot and dry periods [6, 10]. In the Arctic, emissions from wetlands and lakes are projected to increase in response to ‘arctic‐amplification’ driving rapid warming and most likely wetter summer conditions [11, 12]. On the other hand, emissions may decrease in wetland regions that become drier [13].

Efforts to reduce direct anthropogenic sources are underway as part of the Global Methane Pledge, supported in part via observations through the International Methane Emissions Observatory (IMEO). Uncertain emissions pose a challenge for defining baselines and setting reduction targets. This is why the Global Methane Pledge also commits to “moving toward using the highest tier IPCC good practice inventory methodologies, consistent with IPCC guidance, with particular focus on high emission sources, in order to quantify methane emissions”. Current progress on implementing pledged emission reductions is insufficient to achieve climate targets [14]. In addition, with the recent acceleration of global warming, climate‐driven increases of natural emissions could further offset mitigation efforts, raising the atmospheric methane mixing ratio and amplifying climate warming. Both anthropogenic emissions estimated by inventories [15] and natural emissions by empirical and mechanistic models [16] are highly uncertain, which makes the definition of baselines and reduction targets challenging. Moreover, some hotspots of natural emissions are located in remote regions of the Arctic, the interior of Africa, and the Amazon, where very few ground‐based measurements exist due to a combination of technical, environmental, financial, and geopolitical obstacles and where satellite retrievals are challenged by cloudy conditions or low sun‐sensor geometries. Therefore, the current global methane observing systems, including satellites, are insufficient to detect early changes in natural emissions, which may cause warming amplification that threatens to compromise efforts to mitigate anthropogenic emissions of methane.

This is important for the long‐term temperature goal of the Paris Agreement given the role of methane management in limiting overshoot, and the potential for warming‐induced emissions to act as a “headwind” to the notion of pulling down global temperatures after exceeding a peak temperature threshold in overshoots. This is because the goal is not methane reduction for methane reduction's sake but to meet climate/temperature goals. Rogerl and Lamboll [17] in calculating remaining carbon budget estimates assumed circa 50% reductions in anthropogenic methane emissions and ignored those natural methane feedbacks.

In this study, we make the case for a pragmatic, integrated, cost‐effective Global Ecosystem‐Methane Observing System (GEM‐OS) in the coming years, as proposed by Watts et al. [18], and where we focus on expected uncertainty reductions. Whereas IMEO is focused on direct anthropogenic emissions from oil and gas (and expanding to aspects of agriculture and waste), additional ground observations would allow the scientific community and policy makers (1) to obtain more accurate emissions estimates with significantly reduced uncertainties in natural systems, across key sectors, and targeted regions, (2) to better separate anthropogenic from natural methane emissions in regions where they are intertwined, (3) to establish the foundational capabilities to detect trends of emissions at the regional level in order to disentangle progress on anthropogenic emissions reductions from natural system emissions and (4) provide an early warning capability for separately detecting rising natural system emissions. In the following, we review the magnitude, uncertainties, and regional trends of current emissions. Then, we set quantitative goals for reducing the emissions uncertainty in key sectors and for improving the completeness of a GEM‐OS to cover all important sources. We explain how these goals could be achieved by adding targeted ground‐based stations of flux and atmospheric concentration in under‐sampled regions, and by better exploiting existing and upcoming space‐borne measurements. The synergetic use of bottom‐up and top‐down observations is highlighted. Finally, we provide notional cost estimates for the addition of ground‐based measurements in support of a comprehensive GEM‐OS to improve observability and reduce uncertainty.

## 2 Current Magnitude and Trend of Emissions

**FIGURE 1:**
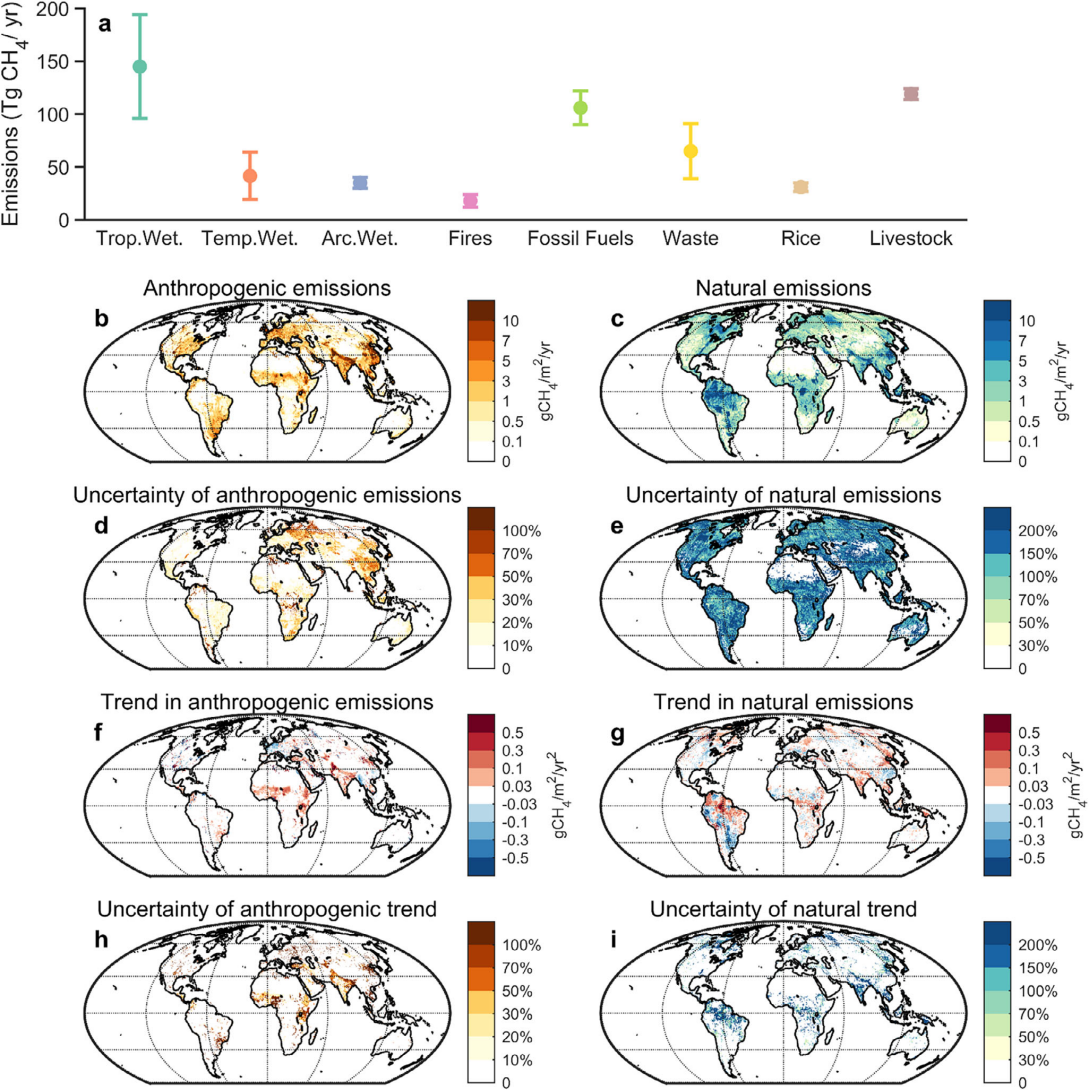
Global methane emissions and their trends from anthropogenic and natural sources. (a) Global methane emissions and their uncertainties as given in Table 1 (b) Total anthropogenic emissions from fossil fuel, waste, rice, and livestock averaged from multiple inventories (Supporting Information) (c) Total natural emissions from wetlands, inland waters, and wildfires from bottom‐up models used in Ciais et al. [20]. (d, e) Current relative uncertainty estimated from the min‐max range of multiple models divided by their mean in each grid cell. (f, g) Current trends from bottom‐up models during the period 2014–2023 were estimated by fitting linear curves to annual emission data. (f, g) Relative uncertainty on the trends estimated from Mann‐Kendall tests on fitted curves and the spread of the trends from individual models (see details in Supporting Information).

Figure 1 displays the average magnitude, trends (including uncertainty), and the spatial distribution of anthropogenic and natural methane emissions at 0.5° for the period 2014–2023. Uncertainty is estimated from the spread among multiple independent bottom‐up gridded inventories for anthropogenic emissions (EDGAR v2024/v2025 and CEDS) and among multiple emission models/datasets for natural emissions (wetlands and fires), using a normalized min–max range approach (Supporting Information).

For natural emissions, boreal and arctic wetlands have the largest relative uncertainty (87%) but a smaller magnitude (20–50 TgCH_4_ year^−1^) than the one of tropical wetlands and temperate wetlands. Tropical wetlands have a relative uncertainty of 76% (range 90–200 TgCH_4_ year^−1^), and wildfires have an uncertainty of 70% (range 14–24 TgCH_4_ year^−1^). Inland waters' natural emissions are not represented in Figure 1 due to the lack of annual gridded estimates from multiple models over the period 2014–2023. They have been estimated by Saunois et al. [19] to have a mean average magnitude of ∼120 TgCH_4_ year^−1^ with a relative uncertainty of about 100%.

Anthropogenic emissions at 0.5° spatial resolution have a maximum rate of 120 gCH_4_ m^−2^ year^−1^ in Northern China (coal mines, landfills) and northern India (rice), high emission rates of 7–9 gCH_4_ m^−2^ year^−1^ in eastern North America (fossil fuels, landfills and livestock) and Europe (landfills and livestock), and lower values in southern Brazil (livestock), northern and eastern Africa (livestock) and in South Africa and Australia (coal mining basins). Across all 0.5° grid cells, the average relative uncertainty of the sum of anthropogenic emissions is 24% (0.02%–300%). Globally, we estimate a relative uncertainty of 30% for fossil emissions (90–122 TgCH_4_ year^−1^), 37% for rice (25–37 TgCH_4_), 30% for livestock (95–129 TgCH_4_), 35% for waste (55–78 TgCH_4_ year^−1^), that is a total uncertainty of 32% for total anthropogenic emissions, consistent with the latest global CH_4_ budget assessment [19]. These sectoral uncertainties were estimated from the spread among multiple independent inventories and models available for each emission sector using a normalized min–max range approach (Supporting Information).

Natural and anthropogenic emissions have emissions intensities per unit area that are comparable in magnitude, from their distributions at 0.5° resolution (Figure 1b,c). However, fossil fuel and landfill emissions are from point sources, which have a larger intensity locally than natural fluxes. Distinct spatial patterns emerge, with regions dominated by wetlands and inland waters with little anthropogenic contributions in central Africa, the Amazon, northern Canada, and western Siberia, regions dominated by anthropogenic emissions in China, the Middle East, Central Asia, Australia, South Africa, and regions where natural and anthropogenic emissions are intertwined such as Northern Sub Saharan Africa (wetlands, livestock), Asia and South‐East Asia (wetlands and rice), Europe (wetlands and livestock), and temperate North America (fossil, wetlands, livestock).

The trends of anthropogenic emissions from 2014 to 2023 exhibit substantial spatial heterogeneity (Figure 1f). Local trends exceed 5% of the decadal mean emissions in 17% of grid cells. Our bottom‐up datasets indicate decreasing anthropogenic emissions in North America, Europe and China from 2014 to 2023 and pronounced increases across northern Africa and Brazil due to livestock, and strong growth in India associated with rice cultivation. Importantly, the trends of natural emissions are positive in 55% of grid cells, largely driven by climate‐induced wetland emissions increases and recent increases in boreal fires. Decreases of natural emissions are predicted in southern Brazil, the Sahel–Sudan region, and in China. Considerable uncertainties remain on these trends, particularly for wetland emissions, as recent modeled increases by bottom‐up models used in Figure 1 are smaller than those inferred from atmospheric data [20]. At a 0.5° resolution, the median uncertainty in the trends is 0.0054 g CH_4_ m^−2^ year^−2^ (up to 0.13 at the 90th percentile) for anthropogenic emissions, and reaches up to 0.080 g CH_4_ m^−2^ year^−2^ (up to 0.45 at the 90th percentile) for natural emissions (Figure 1h,i; Figure S1). These trend uncertainties were estimated from the spread of linear regression slopes across multiple independent inventories and models, using the normalized width of the 95% confidence interval of the estimated trends (see trend calculation details in the Supporting Information). Figure S1 shows maps of trend uncertainties in g CH_4_ m^−2^ year^−2^ instead of in percent as in Figure 1h,i.

Over the period from 2021 to 2030, according to the Global Methane Status Report 2025 [21], the full implementation of Methane Action Plan commitments would reduce anthropogenic emissions by up to 42 TgCH_4_ year^−1^ by 2030 (Figure S2). This 42 TgCH_4_ year^−1^ reduction by 2030 in the Global Methane Status Report for full implementation of Nationally Determined Contributions and the Methane Roadmap Action Programme (M‐RAP) commitments would result in an 8% reduction below 2020 levels.

However, atmospheric inversion results suggest that fossil sources remained stable and that agricultural emissions continued to rise until 2023, yet with inversion uncertainties being larger than the 8% pledged reduction signal [20, 22]. Figures ES 3 and 5 with our projections of anthropogenic emissions based on Nationally Determined Contributions and the Methane Roadmap Action Programme (M‐RAP) commitments indicate that reductions are modeled to occur in the latter half of the decade, with most of the reductions expected from the fossil sector, with smaller reductions in the Agricultural and Waste sectors. These differences in trend are not necessarily inconsistent with atmospheric inversion results up to 2023. They point to the need to detect whether the trend of reductions in all three sectors expected in the latter half of this decade is detectable.

In parallel, wetland and biogenic sources of emissions have increased since the mid‐2000s, consistent with long‐term ^13^C isotopic trends in atmospheric CH_4_ [20, 23, 24]. A recent peak of wetland emissions occurred between 2019 and 2022, during which tropical wetlands had wetter anomalies in Africa and Asia and arctic wetlands experienced wetter and warmer climate conditions [20, 25]. Fire emissions exhibit divergent trends, decreasing in African savannas but increasing in tropical and boreal forests.

## 3 Uncertainties and Observability of Emissions

The current uncertainties reported in Table 1 and displayed in Figure 1c,d are based on the min‐max range of emissions estimated from different bottom‐up models for each sector. They represent the structural or methodological uncertainty in the choices we make in building a model or an inventory. The true real‐world uncertainties are certainly larger than the current uncertainties because some emissions are missed by models or have a true value outside the min‐max range of current bottom‐up model ensembles [26]. The true uncertainty of our estimates is unknown and dominated by systematic errors, mainly model extrapolation errors. One could further split uncertainties into, e.g., ‘known knowns’ (what we show in Figure 1), ‘unknown knowns’ (super emitters, inventory gaps, agriculture variations), ‘known unknowns’ (climate feedbacks, abandoned wells, intermittent venting, coal fires), and ‘unknown unknowns’ (any process not yet discovered or upscaled into regional estimates).

**TABLE 1:** Current best estimates of global methane emissions by major source type, current emission magnitudes and uncertainties, and projected future uncertainty‐reduction targets over 5‐ and 10‐year time horizons. Current magnitudes and uncertainties reflect recent synthesis studies and inventories and highlight key sources of unknown bias and data gaps. Notional uncertainty reductions are based on anticipated improvements in observational coverage, expanded flux measurements, enhanced emission factors and activity data, higher‐resolution land and wetland mapping, and assimilation of satellite Earth observation data and improved process modeling. Priority data collection needs to identify regions, source systems, and measurement strategies required to achieve these reductions.

Source type	Current magnitude/uncertainty Tg CH_4_ year^−1^ / %	Uncertainty target of mean emissions in 5 years (%)	Uncertainty target of mean emissions in 10 years (%)	Priority data collection areas
Wetlands Arctic and Boreal permafrost domain	30–40/66% Range of two recent estimates, Kuhn et al. [65] and RECCAP2 permafrost [66] Unknown bias Lack of flux tower data over 66% of the environmental space Lack of accurate peat and small wetlands maps (which consist of fens, bogs, thermokarst bogs of different ages following permafrost thaw and < 1 km^2^, often smaller)	≈ 40% Uncertainty reduction on bottom‐up data‐driven models is estimated based on the availability of more accurate high‐resolution wetland maps and higher res dynamic open water maps (halved spatial uncertainty), as well as a reduction in spatial representation error of 13% equivalent to adding 5 new flux tower sites [72], and a reduction in spatial representation error of 20% by assimilation of EO‐data and improved model representation of permafrost processes.	≈ 25% Uncertainty reduction based on the availability of more accurate high‐resolution wetland maps (two‐thirds reduction of spatial uncertainty), as well as a reduction in spatial representation error of 19%, equivalent to adding 5 new flux tower sites [72] and a nominal further reduction in representation error of 30% by assimilation of EO‐data as well as improved model representation of permafrost processes.	‐Improved separation of wetland types in maps ‐Improved mapping, monitoring, and modeling of abrupt thaw landforms, including post‐thaw lakes and wetlands ‐Improved monitoring in the Russian Arctic and Boreal zone. ‐Assimilation of EO full‐column data
Temperate Wetlands (30‐60°N)	19–64/131% Min‐max of GCB‐CH4 models Unknown bias Lack of small wetlands maps	76% Calculated using the UPCH4 model as the expected reduction of bias (unknown uncertainty) through the utilization of expanded flux measurements as planned in the FLUXNET‐CH4 V1 and V2 synthesis database (Figure S3) [77].	66% Calculated using the UPCH4 model as the expected reduction of bias (unknown uncertainty) after deploying new monitoring flux tower sites (assume 15 new sites; Figure S3) randomly selected across temperate wetland regions.	New flux towers in temperate wetland complexes + improved mapping of small wetlands and peatland areas, including maps of high‐resolution time‐varying water table depths in non‐inundated wetlands.
Tropical Wetlands (30°S‐30°N)	96–194/76% Min‐max of GCB‐CH4 models Unknown bias Lack of flux tower data over several key wetlands. Lack of accurate peat and small wetlands maps	65% Calculated using the UPCH4 model as the expected reduction of bias (unknown uncertainty) through the utilization of expanded flux measurements as planned in the FLUXNET‐CH4 V1 and V2 synthesis database (Figure S3) [77].	25% Calculated using the UPCH4 model as the expected reduction of bias (unknown uncertainty) after deploying new hypothetical monitoring flux tower sites (assume 15 new sites; Figure S3), including hotspot regions, such as the Amazon, the Congo, and the Sudd wetlands, and constrained small wetland areas	New flux towers in the Amazon, African (Chad, Sudd, Cuvette Centrale) + improved mapping of small wetlands and peatland areas, including maps of high‐resolution time‐varying water table depths in non‐inundated wetlands.
Fires	12–24/70% Min‐max range of GCB‐CH_4_ Unknown bias Up to 30% underestimation from omitted small fires and incorrect emission factors [10]	50% Estimated by assuming small fires and improved emission factors will be included in future emissions datasets, reducing unknown bias	25% Estimated by assuming small fires, improved emission factors, including soil burning, will be part of future emissions datasets, reducing unknown bias	Improved mapping of small fires, fuel loads, combustion completeness, and emission factors per fire type
Rice	27–35/40% Min‐max of recent models (Appendix), consistent with GCB‐CH_4_ models Unknown bias Lack of emission factors/flux data covering relevant management practices. Inaccurate paddy area datasets	30% Estimated by expert judgment based on the difference between current realistic models that include some management and climate effects with accurate paddy area [153] vs. Tier 1 models (assuming more realistic models in the next 5 years).	20% Estimated by expert judgment based on the difference between models that include most management practices and climate effects with accurate paddy area vs. Tier 1 models (assuming more realistic models in the next 19 years).	High‐resolution rice cultivation areas, including paddy and other types, and crop calendars. Meta‐analysis of emission factors and new flux towers to cover key management practices influencing emissions (organic/mineral fertilizer types and doses, residues management, flooding duration, crop varieties)
Livestock^a^	114–124/30% Min‐max range of GCB‐CH4 inventories Unknown bias Lack of space‐ and time‐variation of feed type Lack of regional animal density data, including livestock weight, diet, and distance travelled by animals in grazing systems with seasonal herd migration, e.g., in African drylands. Lack of official activity data for countries and products that do not report to FAO Lack of time variation in livestock production systems	25%^b^ Uncertainty reduction on bottom‐up inventories is estimated by assuming Tier 2 estimates to be more accurate than Tier 1, and that all Tier 1 countries accounting for over 50% of global cumulative emissions, ranked from the highest emitter downward, will move to the more accurate Tier 2 inventories in 5 years. Satellite atmospheric measurements combined with a denser ground network in the tropics would typically bring an error reduction of 30% on tropical livestock emissions and reduce the current bias, as shown by our network design results [43] separating livestock and other emission sources in Figure S4.	24%^b^ Uncertainty reduction on bottom‐up inventories is estimated by assuming Tier 2 estimates to be more accurate than Tier 1, and that all Tier 1 countries, accounting for over 90% of global cumulative emissions, ranked from the highest emitter downward, will move to the more accurate Tier 2 inventories in 5 years.	Data from South Asia, Southeast Asia, and Sub‐Saharan Africa Livestock densities and production systems, including location and animal numbers in dairy confined animal feeding units.
Fossil	106/30% Min‐Max range of recent inventories (Appendix) Unknown bias Global underestimation by omitting ultra‐emitters (≈ 7%^b^) and other unreported fugitive emissions (up to 20%). Regional biases can be positive or negative.	20% Estimated by an emission retrieval uncertainty of 15% for a fraction of 60% of global emitters by leveraging all existing satellite capabilities, plus an uncertainty of 30% (as of today) for the rest of the emitters	14% Estimated by an emission retrieval uncertainty of 10% on a fraction of 80% of global emitters from current and planned satellites, allowing to constrain additional smaller emitters, plus an uncertainty of 30% as of today on the rest of emissions	Increase observability (spatial and temporal coverage) and lower detection thresholds of satellite measurements to capture intermittent leaks and smaller emitters than presently observable.
Waste	≈ 65/80% Unknown bias Omission of landfills and under‐reported emissions (up to 20%). Lack of comprehensive emission factors for wastewater (positive or negative bias of up to 100% depending on technology type)^c^	60% Estimated by an emission retrieval uncertainty of 45% for a fraction of 60% of all landfills, including large landfills with an emission rate higher than 0.2 tCH_4_ hr^−1^ quantified by satellites, smaller landfills remaining at 80% uncertainty. An uncertainty of 80% on wastewater treatment in the absence of comprehensive emission factors and activity data	36% Estimated by an emission retrieval uncertainty of 25% for a fraction of 80% of all landfills, including medium and large landfills with an emission rate higher than 0.1 tCH_4_ hr^−1^ quantified by satellites and ground campaigns, smaller landfills remaining at 80% uncertainty An uncertainty of 60% on wastewater treatment with new emission factors and activity data in the main emitting countries	Mapping of all landfills and management types, mapping of centralized wastewater processing plants, and emission factors from ground campaigns Increase observability (spatial and temporal coverage and lower detection thresholds of satellite measurements.

^a^ Uncertainty reduction for livestock emissions is calculated as the relative difference between Tier 2 and Tier 1 estimates (Tier 2 – Tier 1). All Tier 1 countries accounting for over 50% and 90% of global cumulative emissions, ranked from the highest emitter downward, were assured to move to the (assumed) more accurate Tier 2 inventories, resulting in a 17% and 18% reduction in uncertainty (i.e., with a target uncertainty from the current 30% to 25% and 24%), respectively.

^b^ From Lauvaux et al. [50]

^c^ From a detailed study of >100 wastewater plants in China by Sun et al. [154].

Bottom‐up models could be evaluated against independent observations, which could, in theory, allow us to weight models in an ensemble and reduce the current uncertainty range. But the models often share the same biased input data sources, like wetland area maps, livestock densities, and emission factors, and they cannot be comprehensively evaluated. Intensive observation campaigns including airborne data, tall towers for atmospheric concentrations, and dense flux tower measurements may constrain accurate—close to true—estimates of emissions in a test region, which can be compared with models and provide a snapshot quantification of the true uncertainty. However, such campaigns [27, 28] have been focused on small regions and mainly covered anthropogenic emissions hotspots, with very few campaigns for regional natural sources [29, 30].

Sink pathways are also uncertain. In particular, the magnitude and patterns of the OH sink, which differ between model estimates and their dynamical and photochemical drivers [31, 32], can be partly constrained by observation‐based models [20, 33]. The removal pathway of CH_4_ oxidation in the stratosphere, which also produces water vapor and reactive chemicals that affect climate and ozone, was until now estimated based only on models. Using satellite observations, a recent observationally based estimate [34] showed that models systematically underestimate this loss, which should be incorporated in inversions. The soil CH_4_ sink is usually prescribed to inversions and can now be mapped more accurately from upscaling of local measurements [35] and publicly available global datasets [36]. CH_4_ uptake by upland trees has also been estimated globally [17, 37].

Reducing uncertainty of regional and sectoral emissions requires not only adding targeted local flux measurements in regions or subsystems where the largest uncertainties prevail (Figure 1c,d) but also ensuring completeness in the observability of all emissions, including ‘known unknown’ and ‘unknown known’ sources [38, 39, 40]. Completeness is brought by atmospheric measurements translated by atmospheric inversion models into large‐scale complete emission maps. In inversions, concentrations are inversely projected to a net flux through an atmospheric chemistry‐transport model. In principle, inversions constrained by dense atmospheric measurements and perfect knowledge of atmospheric removal sinks of CH_4_ could allow a reduction of bottom‐up uncertainties and a correction of systematic errors. Indeed, regional inversion models have pointed out biases of bottom‐up models [41, 42]. However, despite their major contribution to constraining methane emissions, current global inversion systems still exhibit substantial spread related to uncertainties in CH_4_ sinks (e.g., OH and chlorine radicals), atmospheric transport representations, and methods used to partition posterior fluxes into emission sectors [41]. In addition, the sparse and uneven spatial distribution of the current atmospheric observation network, particularly in tropical regions, limits the ability of inversions to robustly constrain some regional and sectoral emissions [19]. For these reasons, the addition of new atmospheric measurements in low observation regions drives our uncertainty reduction strategy below.

## 4 Strategies to Reduce Uncertainties, Emission Hotspots, and Diffuse Sources

In this section, we propose achievable uncertainty reduction goals for key methane source types. We show how these targets could be reached by expanding flux measurements to cover ecosystems and processes in places where limited observations exist and by adding atmospheric measurements to ensure the completeness of emission budgets. We adopted a heuristic approach to focus on distinct observational constraints needed for each sector to achieve lower emission uncertainties.

We estimate notional reduced uncertainties on emissions from arctic and boreal permafrost wetlands, temperate, and tropical wetlands (Sections 4.1–4.3) by adding virtual flux towers and calculating the resulting emission bias reduction using the UPCH4 wetland emission model. We estimate reduced uncertainty on rice paddy emissions (Section 4.4) with qualitative analysis adding new flux towers and additional emission factors over a representative set of cultivation practices. For reduced uncertainty on fire emissions (Section 4.5), we consider combining direct atmospheric constraints on fire CO emissions with improved emission ratios of fire CH_4_ to CO emissions. For reduced uncertainty in diffuse emissions from grazing livestock emissions (Section 4.6), which cannot be addressed by adding flux tower measurements, we estimate how uncertainty can be reduced by additional measurements of emission factors across representative livestock production systems and regions to produce improved inventories. To reduce fossil emissions uncertainties, we consider leveraging and increasing the systematic detection of sources using planned satellite constellations to quantify emissions from a larger number of point sources than at present, and to constrain emissions of major fossil extraction basis by regional inversions applied to satellite images (Section 4.7).

For constraining regional and continental scale emissions budgets for all sectors and per sector, we consider adding new atmospheric stations. The focus is on tropical continents where the atmospheric network is critically lacking observations. Uncertainty reductions were calculated for emissions in Africa with a quantitative atmospheric network design model using the LMDz‐INCA inversion system, in which new virtual atmospheric sites measuring the total column of CH_4_ during clear‐sky hours are optimally added to maximize the overall uncertainty reduction. Uncertainty reductions as a function of added atmospheric sites calculated explicitly for Africa are expanded to South America and Southeast Asia by simple scaling.

The problem for sources that can see their uncertainty reduced by adding stations is not that we simply need more stations, but rather how many observing sites are needed to provide the desired information content gain at an accessible cost. The optimal design of atmospheric observing networks depends on both the target gas and the precise question addressed. It can be quantified using atmospheric inversions with information‐theory approaches, network‐performance assessment methods developed in previous CH_4_ and CO_2_ studies [43, 44, 45, 46, 47]. In this study, for Africa, we used a Bayesian inversion model where the a priori uncertainty of each emission source type is set from the spread of the same bottom‐up estimates as in Figure 1, and the posterior uncertainty reduction is calculated analytically by inverting the Jacobian transport matrix calculated by the adjoint of the LMDz‐INCA 3D transport model. However, it does not include atmospheric transport errors [43].

To address the question of where to add new flux towers for reducing the uncertainty of wetlands and rice emissions, we used global empirical flux upscaling models. To address the question of where new atmospheric stations should be added to improve the completeness of emissions and reduce uncertainty on natural and anthropogenic emission budgets of poorly covered regions, we take the case study of a hypothetical denser network of atmospheric stations measuring column CH_4_ to constrain emissions in Africa, an under‐sampled continent with intense wetlands, fires, and livestock emissions. The lessons learned from this case study give us a good basis for recommending a desirable density of new atmospheric measurement sites in other under‐sampled regions. To address the question of how to reduce the uncertainty of fossil fuel and landfill emissions, we consider how constellations of satellite imagers help to detect and quantify a larger number of point sources using local inversion models, such as high‐resolution inversions and plume inversion models, so that a larger global share of those emissions gets constrained. Atmospheric transport errors in local plume inversions reflect uncertainty in local wind magnitude and direction, and plume buoyancy coming from atmospheric stability parameters. Large‐scale transport errors in global inversions include large‐scale wind field errors, including meridional and vertical transport by convection. Our study does not address requirements to improve meteorological data, particularly the use of radiosondes to improve wind patterns and atmospheric transport.

**FIGURE 2:**
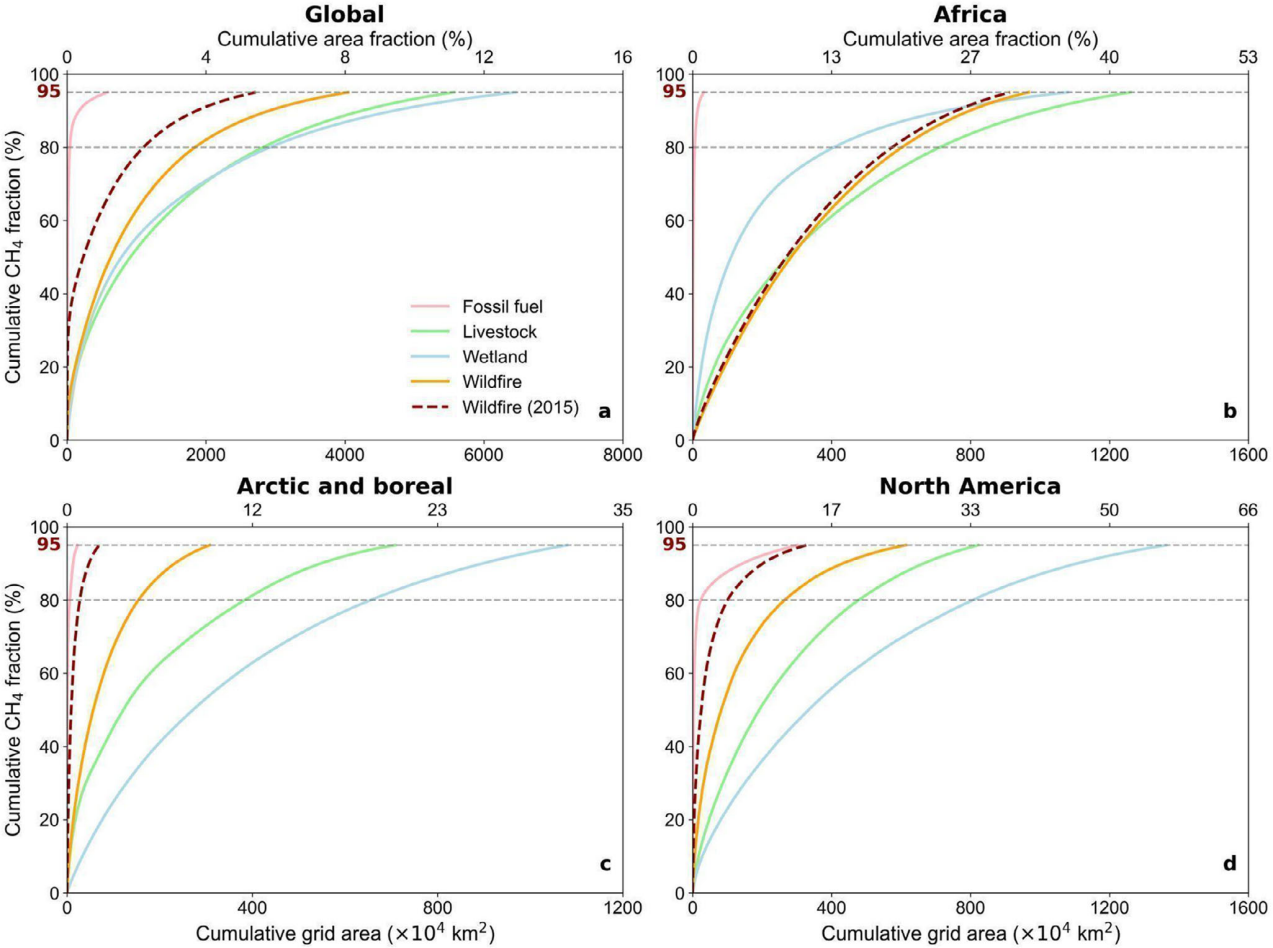
How CH_4_ emissions from different sectors concentrate in a fraction of the grid cells covering selected regions. Cumulative distribution of methane emissions during 2014–2023 from fossil fuels (pink), livestock (green), wetlands (blue), fires mean (yellow), and fire for a single year in 2015. (a) Globe, (b) Arctic and boreal, (c) Africa, and (d) North America. Grid‐level emissions calculated as annual mean during the period 2014–2023 are ranked from the highest to the lowest for each source sector, and their cumulative emission proportions were plotted against the corresponding cumulative grid‐cell areas (bottom x‐axis in km^2^) and cumulative area fraction (top x‐axis in %). Methane emissions from fossil fuel production used EDGAR v2024 and v2025 at 0.1°spatial resolution. Livestock (mean of four datasets) and wetland methane emissions (mean of six datasets) are from Ciais et al. [20] at 0.5° spatial resolution. Fire emissions are the mean of three datasets at 0.5° by 0.5° (GFEDv4.1s, GFEDv5s, and GFAS).

First, we show how emissions are distributed in space for different sectors. For fossil fuel emissions, according to global inventories available at the highest possible resolution of 0.1°, only 1% of the 0.1° grid cells with the highest methane emissions account for 80% of the global total budget (Figure 2). High‐resolution airborne spectrometer data collected during campaigns over the US further indicate that fossil fuel emissions are nearly all from point sources [48]. Oil and gas point sources are intermittent [49, 50], with 40% of the fossil point sources being intermittent leaks in the US [48] and 60% being continuous point sources. In contrast, livestock emissions from grazing and ranching are spatially diffuse, with 60% of the emitting area covering 80% of these emissions at 0.5° resolution (Figure 2). Yet, large dairy confined animal feeding operations concentrate emissions over very small areas similar to continuous fossil point sources, but the distribution of these livestock point sources is not known. Wetland emissions are a bit less diffuse than livestock emissions globally, but there are regional differences (Figure 2). In Africa, wetland emissions concentrate in a few wetland complexes (i.e., Cuvette Centrale, Chad, Sudd) but in northern regions they are more diffuse than livestock emissions [51], reflecting a large area of peatlands (>4 M km^2^). Fire emissions are spatially more concentrated than livestock emissions and less concentrated than fossil emissions (Figure 2). In North America, 3.5% of grid cells account for 80% of the fire emissions on average, against 0.5% in boreal and arctic regions (Figure 2). Other regions with concentrated fire emissions (not shown) are in the Amazon arc of deforestation, Southeast Asia, and woodlands north and south of the Congo forests [52, 53]. In contrast, African savanna fire emissions are spatially widespread and more diffuse than wetlands and livestock emissions (Figure 2). The spatial distribution of fire emissions is not stationary, as shown by the dashed red curves in Figure 2 showing that extreme fires covering 0.8% of the Arctic and boreal area and 4.2% of North America accounted for 80% of total fire emissions in the year 2015. Similar extreme years with high fire emissions over very small areas happened in Australia in 2019 [54], Siberia in 2021 [55], Canada in 2023 [56], and in the Amazon in 2024 [57]. The location and variability of fire emissions are well characterized from satellite active fire counts and burned areas data, but the magnitude of the CH_4_ emissions remains uncertain because of uncertain emission factors and emission ratios [58, 59].

Those differences in the positive skewness of the cumulative spatial distributions of emissions between sectors (Figure 2) call for sector‐specific strategies to reduce uncertainties and improve the completeness of total emissions. For emissions sectors dominated by point sources like fossil fuels (and landfills), the best strategy is to target as many point sources as possible using high‐resolution satellite imagery and to achieve significant uncertainty reductions when a point source emission is measured. For diffuse natural emissions like wetlands, the strategy is to add flux towers representative of under‐sampled ecosystems and processes, and improve the mapping of wetland areas and types, including previously undetected small wetlands [60, 61, 62]. For very diffuse emissions like grazing livestock, the strategy is to improve both activity data on the spatial density and type of ruminants and their emission factors. For diffuse sources, adding ground‐based atmospheric measurement stations in under‐sampled regions, combined with satellites, helps to increase the observability of all sub‐regional emissions and better constrain regional totals. In the next sub‐sections, we propose and justify uncertainty reduction goals that could be reached in the next five and ten years for key sources, and discuss specific observation improvement strategies to achieve those goals. Note that our gap analysis does not cover inland water emissions and low emissions or soil CH_4_ uptake locations, which deserve further studies [63]. We did not include shallow Arctic ocean shelf and permafrost‐capped geologic sources that will affect atmospheric balance beyond terrestrial wetlands [64].

### 4.1 Arctic And Boreal Permafrost Wetlands

Arctic and boreal wetland emissions in the permafrost region have an estimated average magnitude of ∼30 TgCH_4_ year^−1^ and a relative uncertainty of 66%. Magnitude is based on data‐driven upscaling models [65, 66] and uncertainty is calculated by combining the propagative additive errors of (i) the mean of relative uncertainty (min max range/mean) from Kuhn et al. [65] and Hugelius et al. [66] as well as (ii) the relative uncertainty (min max range/mean) between the reported mean CH_4_ fluxes of these same studies (26 and 37 TgCH_4_ year^−1^, respectively). The differences between published emission estimates depend to a large extent on the type of wetland (e.g., also see Ying et al. [67]) and the type of permafrost processes included. For example, the mean arctic‐boreal CH_4_ flux of the bottom‐up models ensemble of the Global Methane Budget (GMB) in Ref. [19] is of 17 TgCH_4_ year^−1^, considerably lower than our data‐driven estimates. Hugelius et al. [66] attributed this bias of global models to a lack of permafrost thaw processes, specifically abrupt thaw and thermokarst in the process‐based models. But they found that combining the GMB ensemble estimates with a separate wetland thermokarst model [68] yields estimates that are broadly consistent with data‐driven model upscaling, as presented in Ramage et al. [69] and more recently by Kuhn et al. [65]. Notwithstanding the lack of permafrost‐specific processes, the GMB models exhibit considerable model spread. Even with the same prescribed wetland areas, the relative model uncertainty is 183% when calculated as the min‐max range of models divided by their mean over the 2000–2020 time period.

Kuhn et al. [65] provide a comprehensive data‐driven assessment of northern CH_4_ fluxes to date, including many different wetland and lake types with representation of relevant permafrost processes. This assessment combines the Boreal–Arctic Wetland and Lake Dataset (BAWLD) geospatial database [70] with a separate flux data synthesis database [71]. Separating the sources of uncertainty reveals that it mostly originates from the spatial maps of wetland types (48%) rather than from uncertainty in modeled flux rates per areal unit (9%). It is important to note, however, that this relatively low estimated uncertainty in modeled flux rates per area unit is based on the assumption that all different wetland types are well covered by existing flux data. Pallandt et al. [72] analyzed the representativeness of the current regional flux towers network and found that existing CH_4_ towers cover only 34% of the environmental envelope. Chamber data that have been collected extensively in this region could be included to determine if model extrapolation uncertainty could be reduced from incomplete spatial representation.

However we note that the current uncertainty of 87% could be reduced to 41% in 5 years and to 23% in 10 years (Table 1) by better leveraging the combined strengths of bottom‐up and top‐down methods, including a halving of the uncertainty related to currently poorly constrained wetland areas from more accurate high‐resolution wetland and water body maps with time variations [73, 74, 75], a reduction in spatial representation error of 13% equivalent to adding five new flux tower sites in uncharted environmental conditions [76] and including assimilation of column CH_4_ concentration data from satellites. We note that the efficacy of assimilating satellite data may be limited, e.g., by transport errors, lack of data during winter, and limited detection abilities over water [76].

### 4.2 Temperate Wetlands

Temperate wetland emissions have an average magnitude of 19–64 TgCH_4_ year^−1^ and a relative uncertainty of 131%, based on the GMB models ensemble min‐max range [19]. Theoretical uncertainty reduction was quantified as the expected decrease in bias achievable through expanded flux observations and continued FLUXNET‐CH_4_ synthesis efforts, calculated by adding virtual sites in the UPCH4 machine learning emission model. We considered two uncertainty reduction scenarios: (1) expansion of the flux tower network to include existing sites in FLUXNET‐CH4‐v1 [77] and new sites planned in FLUXNET‐CH4 v2 (Figure S3), and (2) adding 15 hypothetical new sites randomly selected over temperate wetlands, a selection being repeated 20 times. The two scenarios give projected future uncertainty in 5 and 10 years, respectively. In the first scenario, uncertainty on temperate wetland emissions is projected to reach 76% in 5 years. In the second scenario, uncertainty is projected to reach 66%. Beyond reducing flux uncertainty by adding sites, improving the mapping of temperate wetland extent, type, anthropogenic disturbances, and drought‐to‐flood state, and simulating high‐resolution water table depth variations in non‐inundated temperate peatlands is also necessary.

### 4.3 Tropical Wetlands

Tropical wetland emissions have an average magnitude of 96–194 TgCH_4_ year^−1^ and a relative uncertainty of 76% for, based on the GMB models ensemble min‐max range [19]. Because tropical inundation areas were harmonized in the GMB models, model uncertainty arises from poorly constrained measurements of flux rates per area used to parameterize emissions. A significant additional ‘unknown’ uncertainty comes from limited knowledge of the area occupied by wetlands and peatlands, including small wetlands [78] and peatlands which are not inundated but emit CH_4_ even when the water table is below the surface. To help reduce tropical wetland emission uncertainty, the FLUXNET‐CH_4_ synthesis of flux tower measurements was developed to provide high‐quality, standardized measurements of wetland CH_4_ flux intensity at 30‐min resolution [79]. These datasets are essential for calibrating CH_4_‐related parameters in process‐based biogeochemistry models, including those used in GMB. However, parameter calibration across diverse wetland types using large ensemble perturbation experiments remains challenging because of substantial computational demands and structural limitations in current models. In addition, FLUXNET‐CH_4_ offers limited coverage of tropical wetlands, especially major emitting regions such as the Amazon, Congo, and Sudd. Ongoing synthesis efforts for FLUXNET‐CH_4_ Version 2, along with the integration of eddy‐covariance measurements and chamber observations, are expected to improve data coverage and CH_4_ estimation across tropical wetlands.

We estimated using the UPCH4 model that leveraging the existing FLUXNET‐CH_4_ V1 sites, planned FLUXNET‐CH_4_ V2 sites, and hypothetical new sites (Figure S3) would reduce uncertainties in tropical CH_4_ emissions from 76% to 65% in 5 years relative to using only existing sites in FLUXNET‐CH_4_ V1 [77] (Table 1 and Supporting Information). With the strategic deployment of 15 additional monitoring flux tower sites across key hotspot and un‐sampled tropical regions, including the Amazon, Congo, and Sudd wetlands, uncertainty could be further reduced to reach 25% in 10 years (Table 1). This represents a promising direction for future observational networks and data synthesis efforts, although the work is still underway. Beyond reducing flux uncertainty by adding sites, improving the mapping of wetland extent, type, and drought‐to‐flood state, and simulating high‐resolution water table depth variations in non‐inundated wetlands is also necessary. The uncertainties induced by inaccurate wetland area, types, and the range of water table fluctuation are particularly pronounced in the Amazon Basin, the Congo Basin, and Sub‐Saharan Africa.

Although existing wetland datasets focus on representing saturated or inundated wetlands, unsaturated peatlands with water tables below the surface also emit CH_4_ [80, 81] and remain poorly represented. Water table fluctuations are particularly important for floodplains, and changes in the range of ± 0.5–1 m water table have deep consequences for methane fluxes [82]. Recent constellations of GPS satellites (i.e., CYGNSS) have shown promise to help map tropical wetlands at high temporal and spatial resolution [83, 84]. Emerging satellite missions are expected to significantly reduce wetland‐mapping uncertainties. The Surface Water and Ocean Topography (SWOT) mission can strengthen surface water extent and elevation [85], while the NASA–ISRO Synthetic Aperture Radar (NISAR) mission, with its L‐ and S‐band radars, and the SWOT mission for water extent mapping in regions where there is little or no tree cover over flooded areas, is expected to better detect forested wetland inundation due to improved canopy penetration [86]. Together, these missions will enhance monitoring of wetland extent, water level, and inundation dynamics. Complementary ground observations, such as water‐table depth measurements and field‐based wetland inventories, remain essential for validating and improving satellite‐derived wetland products.

### 4.4 Rice

Rice cultivation emissions have a mean emission of ∼32 TgCH_4_ year^−1^ with a current uncertainty of 40%, estimated from the min‐max range of inventories and process models [20]. Like for wetlands, the uncertainty of rice emissions has a large ‘known unknown’ component related to imperfect knowledge of emission factors per unit area, which depend on management practices such as flooding duration and intermittency, fertilizer type and cultivar varieties. Based on results from recently developed bottom‐up crop models [87], which represent a first attempt to capture the impact of management practices on emissions, we estimated this source uncertainty to be a bias of up to 30% on global emissions.

Reducing the uncertainty of bottom‐up rice emission models involves the collection of improved high‐resolution mapping of activity data, including flooded paddy areas and their temporal variability [88, 89, 90, 91] (http://www.earthstat.org/), rice cultivation types [92], and crop calendars [91]. In addition, the collection of direct emissions estimates (e.g., IMEO's new effort to target rice methane) and new flux tower data to monitor CH_4_ emissions and reduce the extrapolation error of models, and the meta‐analysis of emission factor measurements across a wide range of management practices will help to reduce errors due to ignoring spatial variations of emission intensities [93, 94]. We estimated that the combination of these three approaches will reduce the current uncertainty of 40% down to 30% in the next 5 years, and down to 20% in the next 10 years, based on expert analysis of the differences between advanced bottom up models which include management, atmospheric and climate effects on emissions [87] vs. generic Tier 1 models based on static emission factors which under‐estimate the real world regional diversity of emission rates [89, 95].

### 4.5 Fires

Fire emissions have an average magnitude of 19 TgCH_4_ year^−1^ with a relative uncertainty of 70%, based on the min‐max range of GMB estimates [19] (Table 1). Some ‘known unknowns’ uncertainty arises from the extent of underestimated burned area and underestimated emission factors from incomplete combustion processes, such as from wet forest low‐intensity fires and soil burning, including peat fires. Real‐world fire emissions could be 30% larger than current estimates, according to Ref. [10] based on CO inversions. This low bias can now be estimated and corrected due to new burned area datasets [52, 96] that capture small fires, showing a near doubling of burned areas and resulting in an upward shift of estimated CH_4_ emissions. However, uncertainty on the emission factors still persists. The pathway to reduce this source of uncertainty is to acquire emission factors data for smoldering combustions in forest and peat fires [10], which is achievable by combining field campaigns, analysis of atmospheric records of multiple species including CO, CO_2,_ and CH_4_ [97], and use of collocated satellite column enhancements of CO_2_ and CH_4_ over a range of fire types. In addition, an independent constraint on large‐scale fire CH_4_ emission budgets can be achieved by long‐term inversions of CO fire emissions based on MOPITT [98] and TROPOMI [99, 100] satellites combined with CH_4_ to CO emission ratios like in Ref. [10], and by using diagnostic models of fuel load derived from forest structure remote sensing [101].

Here we estimated that the uncertainty on fire emissions could be reduced from 70% to 50% in the next 5 years, and to 25% in the next 10 years, including the correction of current biases by using methods proposed in recent studies [10, 52, 96] (Table 1) for improving emission factors and adding small fires in emissions models [101]. Improved fire emission for CH_4_ can be derived from independent emissions estimates based on carbon‐monoxide (CO) inversions and a conversion factor from CO to CH4. Zheng et al. [101] derived these conversion factors from 148 field measurements from recent literature (their Table S3). These measurements cover a wide range of different types of fires, resulting in values ranging from 0.009 to 0.085 (g kg^−1^/g kg^−1^). In addition, satellite imagers (e.g., TROPOMI) provide detailed information on fire CH4 plumes, which allows high‐resolution regional inversion models to better constrain regional fire emissions for large events detectable by the satellite. Yet, the use of realistic priors given by improved bottom‐up emission models remains critical to accurately quantify regional CH_4_ fire anomalies from satellite atmospheric measurements.

### 4.6 Livestock

Global livestock emissions, including enteric fermentation and manure management, have an average magnitude of 117 TgCH_4_ year^−1^ for the year ca.2020 based on different inventories from GMB [19]. The relative uncertainty is 30%, based on the min‐max range assessed from uncertainty of the emission factors for the IPCC Tier 1 approach and uncertainties in the parameters used by the IPCC Tier 2 approach [102] as described in the section “Uncertainty Estimates” of Chang et al. [103]. Tier 1 inventories like FAOSTAT [104] estimate emissions using a linear relationship with animal numbers, while some non‐linearity arises in practice due to dependencies of emissions on the total weight of the animals and their diet. Nonlinearities are better captured by Tier 2 models and higher‐tier approaches, and starting in 2026, IMEO is currently planning data collections toward improved Tier 2 models. For inventories employing the IPCC Tier 1 approach, ‘known unknown’ uncertainties come from the fixed emission factor values applied (kg CH_4_ per head of animal), while in reality, regional livestock weight, production, and feed composition vary in space and over time following the intensification trend of livestock husbandry. Additional uncertainties come from activity data, including the livestock numbers, live weight, meat, and milk production. Currently, global estimates mainly rely on the single source of activity data from FAOSTAT (https://www.fao.org/faostat/). It is not possible to assess the overall accuracy of this dataset, as the source data is collected by member countries and supplemented by imputation for non‐reporting countries and products without official data. Furthermore, livestock distribution and its production systems are key for accurate spatial estimates, especially for large countries such as China, India, the United States and Brazil, while gridded estimates currently mostly rely on FAO's Gridded Livestock of the World (GLW; Gilbert et al. [105], https://www.fao.org/livestock‐systems/global‐distributions/en/) and lack the locations of point sources corresponding to dairy confined animal feeding units.

Overall, moving from the IPCC Tier 1 approach that uses average and fixed emission factors to the Tier 2 or higher approaches when possible [102] is expected to reduce the current uncertainty in livestock emissions. First, activity data should be systematically enhanced through routine cross‐validation against national statistical sources to ensure consistency and accuracy. Second, the development of higher‐resolution activity datasets—at the regional scale or finer—is essential, particularly for large countries where substantial spatial heterogeneity exists in livestock husbandry practices. Third, the characterization of livestock production systems and associated feed composition requires regular revision informed by national and regional surveys, feedstuff statistics, and advanced remote‐sensing‐based assessments of grazing biomass, including the continued improvement and updating of datasets such as Herrero et al. [106]. In addition, downscaled livestock distribution maps and production system classifications should be periodically updated using advanced analytical approaches, including machine‐learning methods that integrate national and subnational livestock statistics with relevant economic and biophysical constraints. Regionally, efforts to improve data collection and data refinement should be prioritized in South Asia, Southeast Asia, and Sub‐Saharan Africa, where substantial increases in livestock production are anticipated but observational data remain scarce. Last, for intensive dairy farms point sources, mobile campaigns measuring emissions and emissions factors [107] and high‐resolution satellite or airborne detection [27, 108, 109, 110] retrieving emissions will help to reduce uncertainties.

Here, we assumed that Tier 2 models provide a more accurate estimation of actual emissions than the Tier 1 approach. So that the relative difference between Tier 2 and Tier 1 estimates (Tier 2–Tier 1) serves as a proxy for the potential reduction in model‐driven uncertainty achievable through methodological refinement. By assuming that countries accounting for over 50% of global emissions, ranked from the highest emitter downward, will all move to the more accurate Tier 2 inventories, uncertainty on global emissions could be reduced from 30% currently down to 25% in 5 years. Assuming that countries accounting for over 90% of global emissions would adopt Tier 2 methods, uncertainty would be further reduced down to 24%. The relatively modest reduction in livestock‐emission uncertainty reflects the limited availability of robust emission factors and observational constraints, particularly for tropical livestock production systems with extensive grazing, with long distances travelled by animals increasing per‐head emissions and quality of forage grass impacting emission factors. In view of this relatively modest uncertainty reduction for an important source based on improved bottom‐up activity data and models, top‐down satellite measurements with satellites and denser ground‐based networks may further correct biases of livestock emissions models at the subnational scale. Adding top‐down atmospheric measurement sites is particularly interesting over tropical pastures and rangelands where 41% of livestock emissions occur [103, 111] and where the collection of improved national data for Tier 2 models may take more time than in other regions. As stated above, the principal advantage of top‐down measurements is to provide a more complete observability of diffuse emissions and reduce the biases of bottom‐up inventories.

### 4.7 Fossil Fuel Emissions

The mean of fossil CH_4_ emission is ∼106 TgCH_4_ year^−1^ with a relative uncertainty of 30%, estimated by the min‐max range of three emissions inventories (Supporting Information). The true uncertainty is likely higher because all inventories may underestimate ‘known unknown’ emissions from point sources and sporadic leaks. We have a good knowledge of the location of potentially emitting point sources, but a poor knowledge of their actual emission rates and intermittency, with major leak events being accidental and generally lasting less than a few hours. We argue that the most cost‐effective strategy to reduce the uncertainty on fossil fuel emissions is to improve the observability of the emissions by detecting and quantifying as many point sources as possible, leveraging atmospheric measurements from campaigns using aircraft, unmanned aircraft systems (UAS) [48, 49], and mobile platforms [112, 113] combined with satellite imagery. Field campaigns provide valuable snapshot estimates of emissions of selected point sources (33, 35, 95), but the intermittent nature of leaks makes it difficult to upscale the results of these campaigns into annual budgets.

**FIGURE 3:**
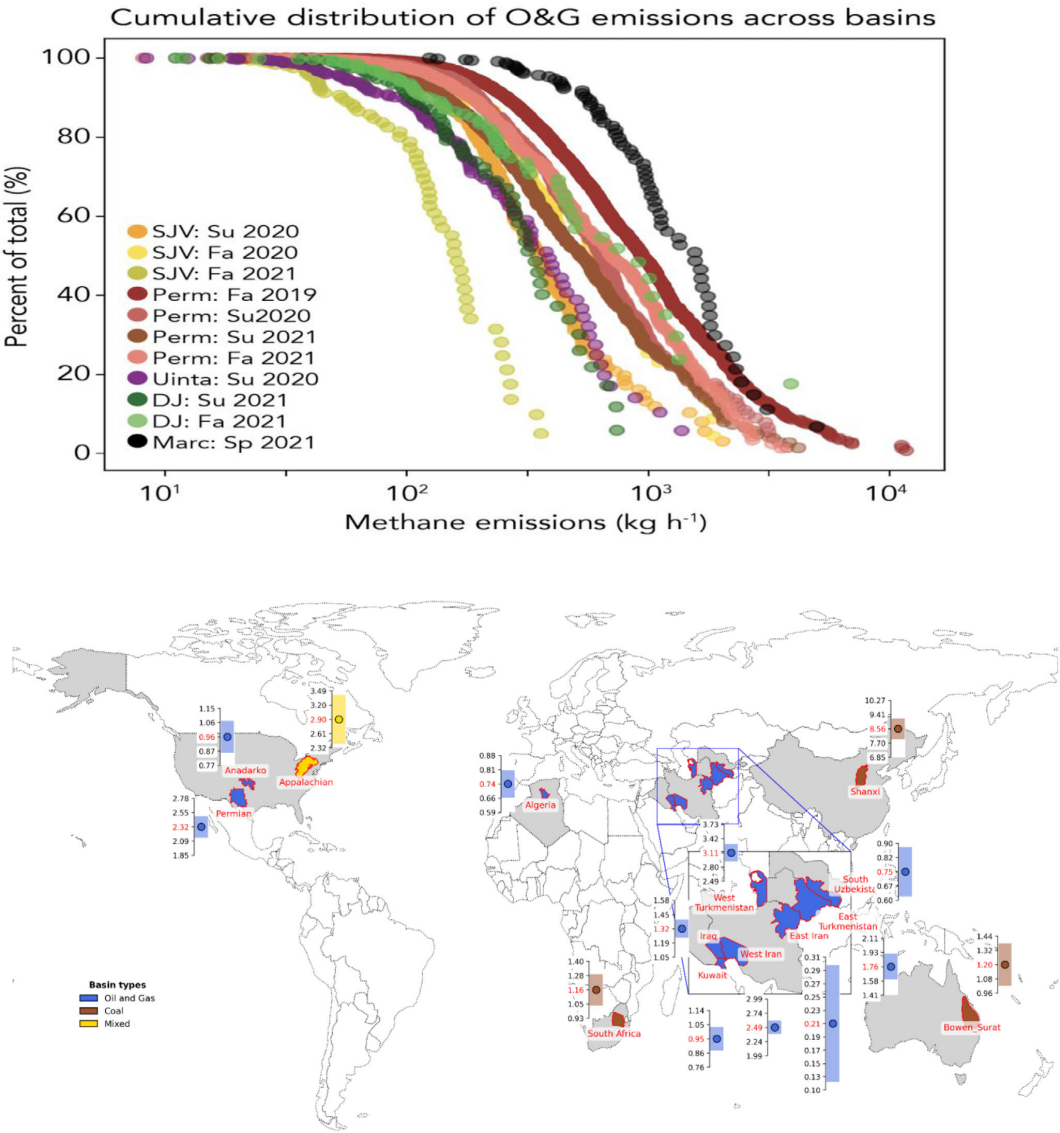
(a) Cumulative distribution of airborne plume emissions quantified from multiple campaigns across different oil and gas extraction basins in the US, from Cusworth et al. [48] (b) Basin‐level fossil CH_4_ emissions quantified by high‐resolution regional TROPOMI flux inversions, from Kayrros Methane Watch data downloaded from https://app.world‐emission.com/detail/regional/ch4/ch4. The basin emissions include oil and gas in the Middle East, Central Asia, Algeria, Permian, Anadarko, and coal mines in Appalachian, Shaanxi (China), Bowen (Australian Bowen), and South Africa. Emissions are presented as the mean from 2019 to 2025 in Tg Ch4/yr. In the y‐axis, the mean value is in red, with values above/below increase/decrease by ± 10%. All basins except East Iran have uncertainties within 20%, with the majority of basins having uncertainties between 5% and 10%.

Satellite imagery measuring column concentrations over point sources (individual sources or local clusters of sources) has the potential to achieve a quasi‐global coverage of onshore emission point sources with snapshot measurements during clear‐sky periods. Yet, the ability of satellite imagers to constrain emissions depends on their spatial resolution and sensitivity to detect an excess of column CH_4_ over an emitting source. For instance, TROPOMI was shown to be sensitive enough to detect large CH_4_ plumes from big leaks with an emission rate > 20 tCH_4_ hr^−1^, which represents only 7% of global fossil emissions [50]. Despite a lack of sensitivity to smaller emission rates for single hotspots, TROPOMI can still measure column CH_4_ excess created by clusters of hotspots within the same extraction basin. For instance, over the Shaanxi region in China, which contains > 1000 mines, Peng et al. [114] exploited TROPOMI images available almost each week with regional inversions to quantify emission rates and emission factors per cluster of mines. Using the same approach, we used high‐resolution inversion results from the Kayrros Methane Watch (https://methanewatch.kayrros.com/) to quantify emissions from 14 fossil fuel extraction basins. The data in Figure 3b show regional inversion results exploiting quasi‐weekly TROPOMI images over each basin. Overall, these TROPOMI regional inversions provide annual emissions estimates over each basin within 20% uncertainty (except East Iran), a significant reduction compared to the current spread of inventories, on the order of 50%. Altogether, the 14 basins shown in Figure 3b represent ∼25% (total of basin means = 28.4 TgCH_4_ year^−1^/ fossil total = 106 TgCH_4_ year^−1^) of fossil total emissions, that is, a reasonable share of the cumulative emission distributions shown in Figure 2.

Satellites such as Sentinel‐2 [115] and Landsat‐8 [116] that were not designed with the purpose of measuring CH_4_ have a sensitivity to atmospheric CH_4_ in their near‐infrared bands, and were shown to bring down the detection limits of hotspots to ≈ 2 tCH_4_ hr^−1^ [117], enabling an increase in the observability of a larger share of cumulative point source emissions. Yet, many emitting points have an emission rate smaller than the limit detected by Sentinel‐2 and Landsat. In recent years, dedicated high resolution commercial instruments such as GHG‐sat (https://www.ghgsat.com), Carbon‐Mapper (https://carbonmapper.org), and the EMIT NASA mission (https://earth.jpl.nasa.gov/emit/data/data‐portal/coverage‐and‐forecasts/) have shown improved capabilities to quantity emitting hotspots with an emission rate as low as a threshold of 50–100 kgCH_4_ hr^−1^, which would typically make it possible to observe a large share of fossil point sources (as shown by comparing this threshold to the distributions in Figure 3 measured by airborne imagery). However, GHG‐sat data are not all publicly available, and with both these satellites being tasked to observe specific regions, it is difficult to predict the share of fossil emissions that could be covered and constrained globally. Further, there is a need to establish measurement standards across platforms [118].

Here, assuming a quasi‐global coverage of fossil emissions point sources above a threshold of 100 kgCH_4_ hr^−1^ as reached by the point source imagers listed above, we estimated, based on limited data from the US (Figure 3a from Ref. [119]) that up to 60% of global onshore emissions could be potentially constrained with an uncertainty of 15% using all available commercial and non‐commercial satellite data. This would result in an uncertainty of 20% on global emissions in the next 5 years, with 40% of non‐observable sources remaining at their current uncertainty of 30% (Table 1). This theoretical uncertainty, based on the assumption that the coverage of current point source detection will be extended in the coming years as satellites continue to acquire data, is supported by practical inversion results. Using all exploitable TROPOMI images with regional inversions, Tibrewal et al. unpublished results shown in Figure 3 for 14 fossil fuel extraction basins (coal, oil, gas, mixed) that comprise nearly one‐quarter of global fossil methane emissions give a mean emission of 28.0 ± 2.7 TgCH_4_ year‐1, that is an aggregated relative uncertainty of 15%. For fossil methane emissions from point sources using the high spatial resolution GHGSat satellite constellation, Jervis et al. [120] showed that exploitable images from the GHGSat satellites detected 8.30 ± 0.24 TgCH_4_ year^−1^ emissions from 3114 emission sites in 2023, which represents 12% of total fossil fuel emissions and an aggregated uncertainty of 3%. Most fossil sources require multiple temporal revisits to capture their annual emissions, given their intermittent nature. Detected oil and gas and coal‐emitting sites above GHGSat's detection limit were found to be emitting 16 and 48% of the time without obvious continental variation by Jervis et al. [120]. From repeated airborne campaigns over the US, Cusworth et al. [48] estimated a persistence of fossil emissions of 30% to 60%. Assuming that the coverage of satellite constellations can reach 80% of onshore emitters with a relative uncertainty of 10% would potentially allow it to reach a theoretical global emission uncertainty of 14% in the next ten years (Table 1). The remaining fossil CH_4_ emissions from offshore facilities and urban distribution networks will remain difficult to observe and may be targeted using mobile platforms in cities [121, 122] and aircraft campaigns [123, 124, 125, 126] in offshore sites.

### 4.8 Solid Waste and Wastewater Emissions

Although the GMP is primarily focused on fossil‐fuel emissions, the waste sector—representing nearly 20% of anthropogenic methane emissions—also constitutes an important component of the initiative. Landfills and wastewater treatment plants emit on average ∼65 TgCH_4_ year^−1^ with a current uncertainty of 80% (Table 1) based on the 95% CI analyzed by the EDGAR database with activity data uncertainty ranges depending on input source and being on the order of 50%–100% with high uncertainties for solid waste, and emission factors uncertainties of around 30% (see table 6 in Ref. [127]). Note that this uncertainty range is larger than the range of GMB inventories, which would give a relative uncertainty of 35%. The true uncertainty is likely larger and could reach up to 100%, as many landfills and wastewater treatment plants are not included in current inventories or their emissions are underestimated by up to 200% for individual sites [128], leading to globally underestimated emissions with different regional underestimations. Like for fossil emissions, we argue that improving the observability of landfills and wastewater treatment hotspots (some of which are monitored through IMEO) is the most effective strategy to reduce current uncertainties. Yet, there are differences between landfills and fossil fuel hotspots: (1) on average, landfills have smaller emission rates, (2) emission rates depend on landfill size but also on how waste is managed, with technologies to collect CH_4_ underground or prevent high anoxic decomposition rates being associated with lower emissions. Not all landfills are properly located in global inventories, with missing sites in urban areas of the south but also in the North, where decommissioned landfills continue to emit CH_4_ after their operational lifetime. Further, flux rates can be significantly skewed.

Reducing the uncertainty on landfill emissions requires a comprehensive inventory of all emitting locations completed by top‐down measurements of high‐emission sites, e.g., from satellite imagers [27]. Maasakkers et al. [129] used the TROPOMI data to detect landfill hotspots and zoomed in with GHGSAT high‐resolution images to quantify the emissions of 4 large landfills with emissions rates ranging from 2 to 29 tCH_4_ hr^−1^. Improving the approach by using more high‐resolution satellite images, Dogniaux et al. [130] estimated emissions from 151 landfills and showed that a detection limit of 0.1 tCH_4_ hr^−1^ was reachable with a typical emission retrieval uncertainty of 45%. Drone technology also offers the possibility to complete landfill measurements [131]. For landfills, we assumed an observability threshold of 0.2 tCH_4_ hr^−1^ and an uncertainty of 45% on each site's emissions retrieval, with a potential coverage of 60% of all landfill sites by multi‐satellite imagery in the next five years. This implies that the current uncertainty of 80% on global landfill emissions could be reduced to 60% in 5 years. With 80% coverage, the same assumption of increased % coverage of landfill sites than the one we assumed for fossil point sources, and a 25% error in each site's emissions retrieval, uncertainty could be further reduced to 36% in 10 years (Table 1).

Centralized wastewater processing plants CH_4_ emissions occur from microbial degradation of organic matter in both liquid streams with high organic loads and sludge. Global emissions from wastewater are estimated at 18 TgCH_4_ year^−1^ [132] with China contributing 1.4 TgCH_4_ year^−1^ [133] with a current relative uncertainty of 50%. The difficulty to constrain waste water emissions is that they are often smaller than satellite detection thresholds and highly variable because CH_4_ production at treatment facilities depends on Chemical Oxygen Demand (COD), pH, nitrate levels, system configuration, and environmental factors such as temperature. The emission factors adopted by the IPCC were based on data from only 15 plants and completely miss this variability. Inventories using IPCC emission factors have, therefore, large biases for the wastewater treatment sub‐sector. Methods such as mobile‐tracer studies and remote‐sensing approaches can capture facility‐wide CH_4_ fluxes [134, 135, 136, 137, 138] more comprehensively, but remain labor‐intensive and have so far been applied to only a small number of facilities. Expanding mobile‐based emission factor measurements across a wider range of environments and treatment configurations is needed to reduce the uncertainty on this anthropogenic source. We assumed, based on recent emission factor measurement campaigns compared to IPCC values, that the global uncertainty on centralized wastewater treatment emissions could be reduced to 30% in 5 years and further down to 20% in 10 years (Table 1).

## 5 Improving the Observability of Emissions With Ground‐Based Atmospheric Measurements

Measurement of atmospheric mole fractions constrains emissions in a different way than bottom‐up models. The mole fractions record the gaseous fluxes integrated by atmospheric transport, making it possible via inverse modelling to retrieve a set of fluxes that best fit the available atmospheric observations. Atmospheric inversion is an “ill‐constrained” problem because we attempt to track methane sources across millions of grid cells using only a limited number of measurement points. To solve this problem, most researchers use a Bayesian approach. This method starts with an “a priori” estimate (a best guess based on existing data and uncertainty levels) and then refines that estimate by incorporating real‐world atmospheric measurements. There is a significant spatial mismatch between the location of ground‐based stations and regions of high emission uncertainty. Because existing monitoring networks have very few stations in the Arctic and tropical continents, it remains virtually “blind” to emissions from these regions, although satellites do provide constraints on these regions subject to caveats.

Satellites complement sparse ground‐based atmospheric networks with their global coverage. Specific parts of the electromagnetic spectrum that they can measure are sensitive to the column‐average concentration of CH_4_, while surface emissions mainly generate concentration gradients in the boundary layer. As a consequence, the spatial and temporal gradients in the satellite CH_4_ column retrievals are smaller than the gradients in the measurements made by surface stations in the boundary layer. Satellites offer the unique advantage of covering remote regions where there is no station, thus complementing the surface network. Satellite data are, however, prone to systematic errors in the retrieval process of inferring column CH_4_ concentration, using radiative transfer models [139].

For satellites to constrain global and regional emissions budgets, accurate measurements are needed with as little regional bias as possible, such as those achieved from GOSAT products with a random error on the order of 13 ppb [140, 141]. Imagers like TROPOMI offer a better sampling of the atmosphere but have shown regional biases which translate into biases of global inversions, although retrieval improvements regularly improve the quality of data (i.e., reduce bias, increase precision and accuracy). Satellites using passive spectrometers also provide no data in cloudy areas, and no data in the high latitudes from the Autumn to Spring equinox, which hinders their capability for constraining some sources like wetlands in equatorial regions, anthropogenic emissions in Southern China, Europe, and in winter in high latitude regions, although those emissions are locally small at that time of the year. For emissions hotspots such as in fossil fuel extraction basins, high‐resolution images are needed in order to better detect and quantify emissions using, for instance, plume dispersion models or regional inversions.

**FIGURE 4:**
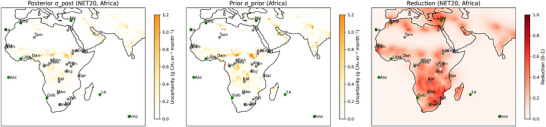
Simulated uncertainty reduction on emissions from all sectors in Africa using 20 new CH_4_ atmospheric measurement sites from a network of EM27/SUN instruments measuring total column CH_4_ from the ground. The theoretical uncertainty was calculated using the atmospheric transport matrix of the LMDZ global tracer transport model at 1° resolution applied to the current (prior) uncertainty map given by the min‐max range of the bottom‐up models as displayed in figure 1. The map of uncertainty reduction on the right is calculated as 1 – Posterior 1‐sigma uncertainty / Prior 1‐sigma uncertainty, so that a value close to 1 is for a very well constrained emission after adding the 25 sites. This optimistic result assumes perfect atmospheric transport simulation, isotropic prior error correlations with an e‐folding length of 500 km over land, and no sub‐monthly variations in the emissions. Prior error variances are derived from the emission maps shown in figure 1c, d.

Simulating how to increase existing ground‐based atmospheric networks to create an optimal GEM‐OS is beyond the scope of this study. Further, logistics, costs, political barriers in the real world, and the existence of ‘unknown unknown’ emissions yet to be discovered limit the value of such a theoretical calculation of the optimal placement of new stations. Yet, we provide here a formal uncertainty reduction estimate of all‐sectors emissions over the African continent by deploying a new network of ground‐based atmospheric stations. The lessons from this case study give us some guidance on the number of new measurement sites needed to achieve a more complete observability of all sources with a significant error reduction in other continents, although large‐scale atmospheric transport patterns vary from one continent to another. As it is challenging to build in the next decade many new in situ atmospheric observatories such as the ATTO tall tower in the Amazon due to their high unit costs ($10 M investment and $300k running cost per year), we tested the alternative deployment of cheaper upward looking column CH_4_ automated ground‐based instruments such as EM27/SUN integrated into a weatherproof casing, which have the advantage of lower unit costs ($300k), require only power with low maintenance and are therefore suitable to operate in remote environments [142, 143]. Simulations of the theoretical uncertainty reduction on total CH_4_ emissions from Africa using a network of 20 EM27/SUN are shown in Figure 4, showing an uncertainty reduction of 85% in January and of 86% in July compared to the current uncertainty [43]. The uncertainty reduction for each sector is shown in Figure S4. The inversion experiment relies on a Bayesian observing‐system simulation framework in which hypothetical EM27/SUN stations are incrementally added and optimized according to their information gain on posterior CH4 emissions. The uncertainty reduction is quantified using atmospheric transport sensitivities and prior emission uncertainties while accounting for redundancy between nearby stations. Separate optimization experiments were performed for total, wetland, and anthropogenic emissions, highlighting the larger network density required to constrain spatially diffuse anthropogenic sources. Scaling this result by the ratio of emission to number of instruments to other tropical continents suggests that tropical methane emissions, where the uncertainty is the largest (Figure 1), could be constrained effectively with up to 50 instruments in South America (20), Africa (20), and Southeast Asia (10). EM27/SUN instruments can be deployed in most regions, including remote environments, provided a stable power supply is available, and recent deployments in South America demonstrate the practical feasibility of such networks. However, this approach is less suitable for Arctic regions because of limited sunlight during winter months and challenging meteorological conditions. As the accuracy of inversions still relies on the quality of their priors, such a denser tropical network will still benefit from reduced uncertainties of bottom‐up models for wetlands, livestock, and fires. The role of atmospheric measurements is to ensure that there is a completeness of all emissions being observable and to correct unknown biases of bottom‐up models at the regional scale. We added in the last column (Table 2) an indicative estimate of the costs, including acquisition, deployment, and yearly operations of flux towers and ground‐based EM27/SUN atmospheric column measurements at unit costs with the typical number of new units to achieve the notional uncertainty reduction goals.

**TABLE 2:** Costs of adding new atmospheric measurement sites (total column) and eddy covariance flux towers in the most uncertain emission regions, as estimated in this study for notional uncertainty reductions reported in Table 1, based on current instrument and maintenance costs in the field. Note that gaps in inland waters emissions are not considered here and that flux towers here focus on methane‐producing systems, whereas additional towers would be needed in upland systems to measure methane sinks.

Type	Number of units	Acquisition and installation cost per unit (1000 US$)	Total acquisition and installation cost	Maintenance cost per site per year	Total Maintenance cost during 5 years
EM27/SUN column measurement sites in the tropics	20 Africa, 20 S. America, and 10 S.E. Asia	300	15 000	25	6250
Flux towers in wetlands not covered by current sites	5 Arctic, 15 Tropics, and 5 Rice	200	5000	100	12 500

Isotopes are extremely valuable to constrain the global mix of source types and global trends of each source type [20, 144]. Note, however, that microbial sources constrained by atmospheric ^13^C trends include the sum of livestock, waste, and wetlands emissions, which cannot be further separated. Deuterium and ^13^C atmospheric measurements used together can better separate fossil, fire, and wetland sources [145]. However, a large number of flask stations measuring isotopes would be needed to constrain these source types at the regional scale, which remains a limitation in global inversions [146].

## 6 Top Down and Bottom Up Reinforce Each Other to Reduce Uncertainty

We emphasize that, in any model–data integration framework— sequential or not—the characterization of data uncertainty is as crucial as the data themselves for shaping the final outcome [147]. We argue that a statistically transparent approach is essential for combining bottom‐up and inversion‐based estimates of CH_4_ emissions. Extending the discussion of Enting et al. [148], we concentrate on elements most pertinent to narrowing uncertainties in regions of pronounced methane emissions. A key point is that different observing systems and modeling strategies carry distinct patterns of uncertainty, which interact in consequential ways when merged. Atmospheric concentration measurements used in inversions aggregate signals from numerous processes and broad source areas, so the combined flux that explains the observed concentrations is usually well constrained. This aggregation naturally induces negative correlations among regional emissions estimates in the inversion posterior fluxes.

In contrast, the upscaling of point‐based measurements such as flux towers and flux chambers frameworks like UPCH4 [149]— tends to carry positively correlated errors between predicted emissions on gridded upscaling emission maps. Similar measurement limitations, shared environmental biases, or methodological assumptions often propagate across sites, meaning that an error present at one location is likely to appear elsewhere. In the schematic adapted from Enting et al. [148], the combined constraint (represented by the overlap of the two uncertainty ellipses in Figure 4 of Enting et al. [148]) depends on the contrasting correlation structures of the two approaches; this combined constraint is narrower than what either method could achieve independently.

These differences also explain scale‐dependent behavior in uncertainty. For bottom‐up approaches, uncertainty usually expands when moving from site‐level estimates to regional or global fluxes, and positive correlations across regions become more pronounced. For inversions, the situation is almost the reverse: global methane emissions are constrained by the observed atmospheric growth rate, and provided that uncertainties in the OH sink can be estimated, global totals have smaller relative uncertainty than regional fluxes. Because the total global source strength is fixed by atmospheric trends, inversions inevitably generate negatively correlated regional flux adjustments—an effect well recognized from analogous CO_2_ inversion studies—although full posterior error covariance matrices are seldom reported.

These negative correlations should be viewed as advantageous rather than problematic, as they complement the positive error correlations inherent in bottom‐up products. When combined appropriately, they produce a stronger joint constraint on regional CH_4_ budgets, again analogous to the intersection of the two ellipses in the schematic figure. To enable such dual‐constraint analyses, we recommend that inversion teams routinely release posterior error standard deviations at relevant space and time scales (which implies that spatial and temporal correlations are accounted for). For bottom‐up modeling groups, we recommend that they provide analogous information derived from parameter‐ensemble simulations and, where possible, multiple alternative upscaling strategies (including different machine‐learning predictors).

## 7 Challenges and Opportunities, The Way Forward

Rather than reconciling top‐down and bottom‐up methodologies, our vision to extend on the work of IMEO to meet the three objectives of a Global Methane Observing System (GEM‐OS) via strategic ground measurement expansion (i) substantially lower uncertainty in natural systems and key sectoral and regional emission estimates, (ii) robust separation of anthropogenic and natural fluxes where they co‐occur, and (iii) trend detection at regional scales to verify mitigation progress while providing early warning of emerging natural feedbacks, is broader in that it is to integrate top down and bottom‐up observations and models to bring a dual constraint on estimating emissions and removals to support high resolution regional monitoring and trend attribution and monitor the effectiveness of mitigation efforts to curve down anthropogenic emissions.

Top‐down methods quantify regional budgets at resolutions that approach 1° globally today, with targeted finer‐grain resolution quantification in key locations (e.g., high‐priority oil and gas). In many locations, however, there are various challenges associated with isolating individual sectors. Bottom‐up approaches allow us to obtain a further refined attribution and mapping of different sub‐sectors, wetland types, etc.; however, with considerable uncertainty as the physical representation of these processes is data under‐constrained. An integrated workflow would work toward enabling target conservation priorities and mitigation priorities by focusing on hot spots and moving to higher‐resolution regional emissions estimates.

One of the key remaining challenges involves maintaining greenhouse gas observations across scales, sustained for long time periods, and following globally accepted calibration or data processing standards [150]. Currently, our existing GHG observing networks are the result of a combination of research and quasi‐operational investments that provide valuable information yet have led to temporal inconsistencies and geographic gaps. A full GMOS network, combining existing flask measurements, balloons, flux towers, tall towers, research and commercial aircraft, and satellite missions, would include significant initial investments (i.e., satellite costs are $200‐300 M for a wide‐swath instrument and $100 M for launch) and substantial operational and maintenance costs involving the cooperation of multiple institutions around the globe. Lower cost options to measure CH_4_ fluxes from flux towers may also be available in the future, using other micrometeorological techniques like the flux gradient or modified Bowen ratio that have been applied successfully to fluxes of N_2_O in agricultural settings [151]. The additional minimum eddy covariance towers and ground‐based total column instruments (EM27/SUN) sites to be added in current blind spots of emissions where the uncertainty is the largest in order to achieve the notional targets of Table 1 are listed in Table 2, and would cost only 20 M$ for installation and ≈ 20M€ for 5 years of operation. A more radical idea would be to introduce adaptive observations in regions where early changes are detected, similar to the ideas explored by Craig Bishop in NWP some 25 years ago [152].

The World Meteorological Organization's effort to establish a Global Greenhouse Gas Watch (G3W) program could potentially serve as a clearing house for establishing calibration and data processing standards, integration of observations and models, and providing actionable greenhouse gas emission and removal data with low latency. Such coordination would help reduce the notional uncertainties outlined in the Table through integration, collaboration, and leveraging the comprehensive research and operational communities' capabilities in observations, modeling, and reporting (to National Greenhouse Gas Inventories).

## Author Contributions

**F. Li**: conceptualization, methodology, software, validation, formal analysis, investigation, data curation, writing – original draft, writing – review and editing. **R. Jackson**: writing – review and editing. **A. Hoyt**: writing – review and editing. **G. Hugelius**: conceptualization, methodology, investigation, writing – original draft, writing – review and editing. **Q. Zhu**: methodology, software, visualization, formal analysis, investigation, data curation, writing – original draft, writing – review and editing. **J. Chang**: methodology, investigation, data curation, writing – original draft, writing – review and editing. **K. Arndt**: conceptualization, methodology, writing – review and editing. **R. Varner**: writing – review and editing. **K. Tibrewal**: writing – review and editing, data curation. **F. Chevallier**: methodology, software, formal analysis, investigation, writing – review and editing, supervision. **H. Li**: writing – review and editing. **P. Ciais**: writing – original draft, writing – review and editing, conceptualization, methodology, investigation, resources, data curation, visualization, supervision. **J. Watts**: writing – review and editing. **N. Besic**: writing – review and editing. **H. Cadillo‐quiroz**: conceptualization, methodology, writing – review and editing. **S. Peng**: conceptualization, methodology, formal analysis, investigation, resources, data curation, writing – review and editing, writing – original draft, visualization. **K. Yuan**: methodology, software, validation, formal analysis, investigation, writing – review and editing. **B. Buma**: writing – review and editing. **Y. Cai**: writing – review and editing. **D. Monteverde**: conceptualization, funding acquisition, writing – review and editing. **S. Malone**: writing – review and editing. **R. Vargas**: writing – review and editing. **E. Euskirchen**: writing – review and editing. **S. Natali**: writing – review and editing, conceptualization. **C. Rey‐sanchez**: writing – review and editing. **B. Poulter**: conceptualization, methodology, investigation, resources, writing – original draft, writing – review and editing. **P. I. Palmer**: writing – review and editing. **M. Ramonet**: writing – review and editing. **Z. Zhang**: writing – review and editing, visualization.

## Conflicts of Interest

The authors declare no conflicts of interest.

## Supporting information




**Supporting File**: advs76624‐sup‐0001‐SuppMat.docx.

## Data Availability

The data that support the findings of this study are available from the corresponding author upon reasonable request.
