# Supplementary material for: A Global Methane Observation System to Reduce Uncertainty for Anthropogenic and Natural Sources and Sinks for Detecting and Attributing Climate Feedbacks
Source: Adv Sci (Weinh). 2026 Jul 29:e76624. Online ahead of print. doi: 10.1002/advs.76624 (PMC13418512; doi:10.1002/advs.76624)
Supplement: Supplementary file 1 — Supporting File: advs76624‐sup‐0001‐SuppMat.docx. [file ADVS-9999-e76624-s001.docx]

Supplementary Materials for

**A global methane observation system to reduce uncertainty for anthropogenic and natural sources and sinks for detecting and attributing climate feedbacks**

P. Ciais^1^, S. Peng^2^, J. Chang^3^, F. Li^4^, Q. Zhu^5^, K. Yuan^6^, G. Hugelius^7,8^, H. Li^1^, Y. Cai², F. Chevallier^1^, K. Tibrewal^1^, E.A. Kort^9^, K. Arndt^10^, J. Watts^10^, B. Buma^11^, N. Besic^12^, P. I. Palmer^13^, H. Cadillo-Quiroz^14,15^, E. Euskirchen^16^, M.J. Gondwe^17^, A. Hoyt⁴, R. Jackson⁴, S. Malone^18^, D. Monteverde^19^, S. Natali¹⁰, M. Ramonet¹, C. Rey-Sanchez^20^, L.B. Sagang^21,22^, E.A.G. Schuur^23^, R. Vargas^14^, R. Varner^24,25^, Z. Zhang^26^, B. Poulter^19^

1. Laboratoire des Sciences du Climat et de l’Environnement, CEA CNRS UVSQ, Centre Orme des Merisiers, 91191 Gif sur Yvette Cedex
2. Sino-French Institute for Earth System Science, College of Urban and Environmental Sciences, and Laboratory for Earth Surface Processes, Peking University, Beijing, China
3. College of Environmental and Resource Sciences, Zhejiang University, Hangzhou, China
4. Doerr School of Sustainability, Department of Earth System Science, Stanford, CA, USA
5. Climate and Ecosystem Sciences Division, Climate Sciences Department, Lawrence Berkeley National Laboratory, Berkeley, CA, USA
6. Department of Earth and Atmospheric Sciences, University of Houston, Houston, TX, USA
7. Department of Physical Geography, Stockholm University, Stockholm, Sweden, Bolin Centre for Climate Research,
8. Stockholm University, Stockholm, Sweden,
9. University of Michigan, Ann Arbor MI 48109, USA
10. Woodwell Climate Research Center, Falmouth, MA 02540 USA
11. Environmental Defense Fund, Boulder, CO 80302, USA
12. IGN, ENSG, Laboratoire d'inventaire forestier (LIF), 54000 Nancy, France
13. National Centre for Earth Observation, University of Edinburgh, Edinburgh, UK
14. School of Life Sciences, Arizona State University, Tempe, AZ, USA
15. Biodesign Institute, Arizona State University,Tempe, AZ, USA
16. University of Alaska Fairbanks, Institute of Arctic Biology, Fairbanks, AK, USA
17. Okavango Research Institute, University of Botswana, Maun, Botswana
18. Yale School of the Environment, Yale University, New Haven, CT, 06511, USA
19. Spark Climate Solutions, San Francisco, CA, USA
20. Department of Marine Earth and Atmospheric Sciences. North Carolina State University, Raleigh, NC, USA
21. Institute of the Environment and Sustainability, University of California, Los Angeles, CA, USA
22. Ctrees, Pasadena, CA, USA
23. Center for Ecosystem Science and Society, Northern Arizona University, Flagstaff, AZ USA
24. Department of Earth Sciences and Institute for the Study of Earth, Oceans and Space, University of New Hampshire, Durham, NH USA
25. Department of Geological Sciences and Bolin Centre for Climate Research, Stockholm University, Stockholm, Sweden
26. National Tibetan Plateau Data Center, State Key Laboratory of Tibetan Plateau Earth System, Environment and Resources (TPESER), Institute of Tibetan Plateau Research, Chinese Academy of Sciences, China

# Datasets used to map current emissions and their uncertainties

For anthropogenic emissions (fossil, rice, livestock) we collected three widely used and updated anthropogenic methane emissions datasets, including EDGAR v2024 and v2025 (<https://edgar.jrc.ec.europa.eu/emissions_data_and_maps>, 0.1° spatial resolution), and CEDS v_2025_04_18 (<https://github.com/JGCRI/CEDS>, 0.5°x0.5° spatial resolution). The average distribution of emissions from these three datasets during the period 2014-2023 is given in Figure 1b. For the relative uncertainty of anthropogenic emissions shown in Figure 1d, we first calculated the uncertainty range of maximum minus minimum emissions across the three datasets, then normalized it by the average emission, i.e., (max − min) / mean.

The magnitude and uncertainty of natural methane emissions including wetlands, inland waters, and wildfires are considered here. Gridded wetlands emissions of four models including three process-based models (ORCHIDEE, LPJ-EOSIM, and DLEM) and one upscaled from FLUXNET-CH4 by machine learning method (UPCH4) are from Ciais *et al.* [1] (these four datasets have 0.5°x0.5° spatial resolution) covering the period 2014-2023. Inland water emissions are only from one model (DLEM), thus only considered in the magnitude of natural emissions, but not for the uncertainty estimates. Wildfire emissions including GFEDv4 and v5 (<https://www.globalfiredata.org/>), and GFAS (<https://ads.atmosphere.copernicus.eu/datasets/cams-global-fire-emissions-gfas?tab=overview>) are collected for the magnitude and uncertainty estimates.

The uncertainty of natural emissions is the sum of the min-max range of wetlands emissions (four models) and of fires emissions (three datasets). The relative uncertainty of natural emissions shown in Figure 1e is the uncertainty range of natural emissions (wetlands and fires) normalized by their average emissions (wetlands and fires). Note that uncertainty of inland water is not included in Figure 1e.

# Emission trends and trends uncertainties

The trends of anthropogenic and natural emissions are estimated as the slope of linear regression of the emission time series for each grid cell during 2014-2023. For the trend uncertainty, the 95% confidence interval of the regression slope with available time series (e.g., three time series for anthropogenic emissions, and 12 times series from four wetlands emissions and three fires emissions) was calculated for each grid cell, and uncertainty was defined as the range of the confidence interval (upper bound minus lower bound) normalized by the mean trends. The relative uncertainty of emissions trends displayed in Figure 1h, is estimated by adding the uncertainty of the trends of each bottom up model obtained from Mann-Kendall tests [2, 3] and the min-max range of the trends across different models. In addition to trends uncertainties in percent shown in the main text, we present in Fig S1 the trends uncertainty in emissions units.

#
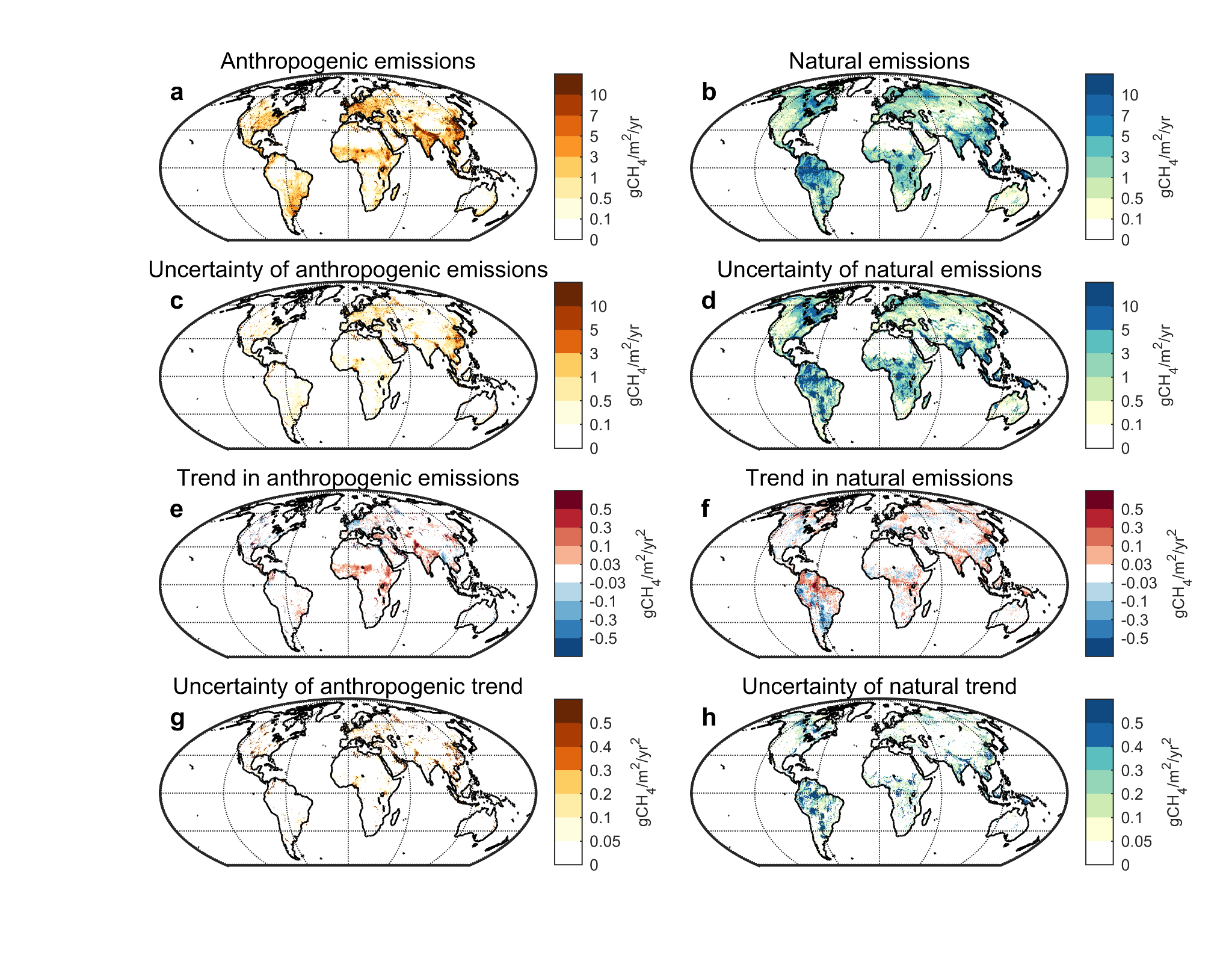


Fig S1. same as Fig 1 in main text but uncertainty on trends are reported in gCH_4_ m^-2^ yr^-1^.

# Emission trends projections

We used the Global Methane Pledge endorsed anthropogenic emissions reductions, assuming that all emission reductions will be implemented by 2035, and a more ambitious scenario, in which all countries will endorse and implement the Global Methane Pledge. For climate induced impacts on natural wetland emissions, we use the results from Zhang *et al.* [4] for two scenarios (RCP2.6 and RCP8.5). The results are shown in Fig. S2.


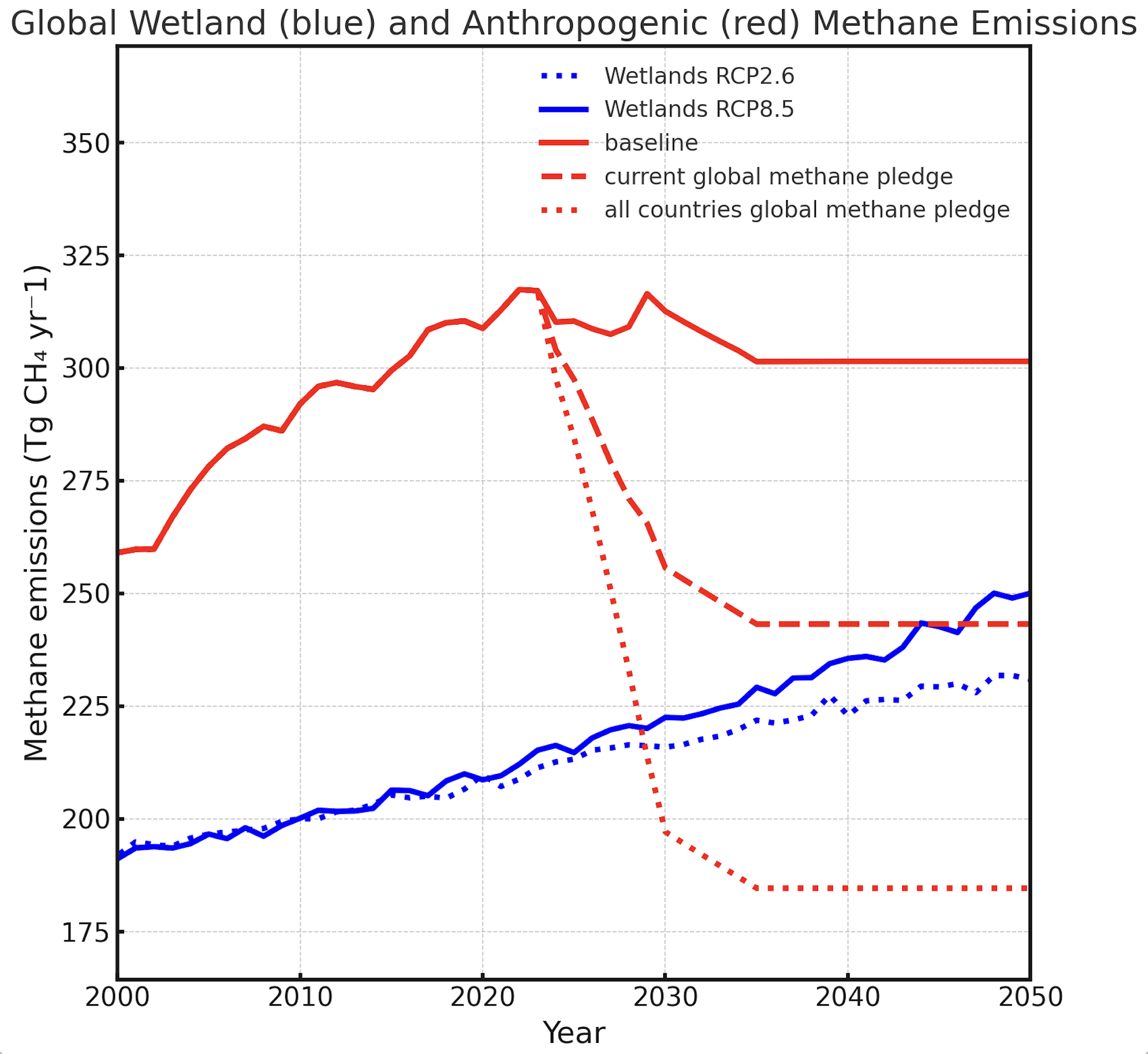


Fig S2. Future emission projections for two Global Methane Pledge endorsement scenarios (all current countries who have endorsed the pledge - dashed / all countries - dotted). The baseline is for RCP4.5 emissions. For climate induced impacts on natural wetland emissions, results from Zhang *et al.* [4] with the LPJ model are shown for two climate scenarios.

# Simulated uncertainty reduction in tropical wetland emissions by adding flux towers

Uncertainty reduction in the tropics is quantified as the expected decrease in bias achievable through expanded flux observations and continued FLUXNET-CH_4_ synthesis efforts. We compared upscaled wetland CH₄ emissions derived from (i) FLUXNET-CH4 V1.0 sites alone (Case 1: baseline) and (ii) an expanded dataset combining ongoing FLUXNET-CH4 V2.0 sites with chamber measurements synthesized in Yuan *et al.* [5] (Case 2: enhanced monitoring network). The reduction in bias between Cases 1 and 2 is interpreted as proxy for uncertainty reduction over the next five years, assuming optimal use of case 2’s data through model–data fusion. To assess future uncertainty reduction beyond current datasets, we estimated the potential bias reduction achievable by deploying 15 new (hypothetical) flux tower sites across representative tropical wetland hotspots [6], including the Amazon, Congo, and Sudd regions. Incorporating these additional sites with the FLUXNET-CH4 V2.0 and chamber datasets, we produced a third upscaled estimate (Case 3: future monitoring network). The bias reduction between Cases 2 and 3 is interpreted as a proxy for uncertainty reduction over the next ten years.

# Current, planned and hypothetical flux towers locations over wetland ecosystems used in the uncertainty reduction calculation of wetland emissions with the UPCH4 model


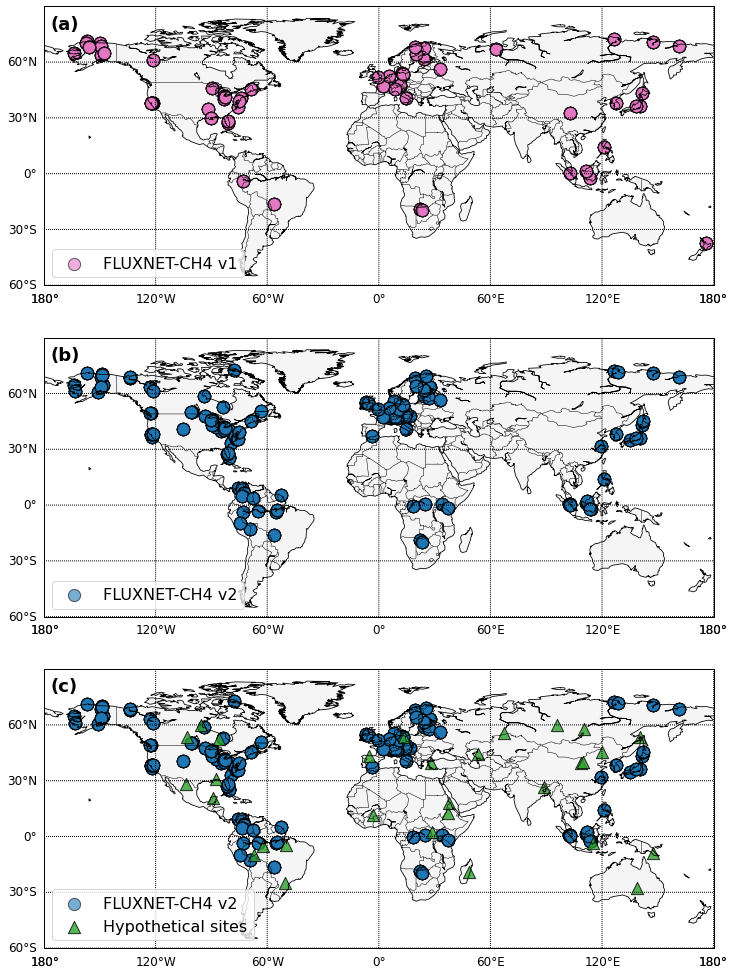


**Fig S3.** Maps of wetland eddy covariance monitoring sites used to quantify uncertainty reduction as the monitoring network expands (Table 1). (a) Current FLUXNET-CH4 v1 network of existing sites. (b) Monitoring network in 5 years, they include possible FLUXNET-CH4 v2 network with newly established sites. Note, this is an ongoing effort. (c) Monitoring network in 10 years, they include hypothetical future monitoring sites sampled from wetland grid cells. Hypothetical sites are drawn from 0.5° × 0.5° grid cells with >1% wetland fraction across temperate and tropical regions. To estimate future uncertainty reduction in regional wetland CH_4_ emission budgets, hypothetical site selection is randomized and repeated 20 times; one realization is shown here.

# Simulated uncertainty reduction in African emissions by adding ten instruments measuring total column CH_4_ mixing ratio during clear sky optimally placed to maximize uncertainty reduction for all sources

Fig. S4. displays the theoretical uncertainty reduction (1 - posterior / prior uncertainty) in the network design study for Africa of Li *et al*. [7]. The uncertainty reduction is shown here for ten additional stations for total emissions, fires, wetlands and anthropogenic emissions (livestock, fossil, waste).


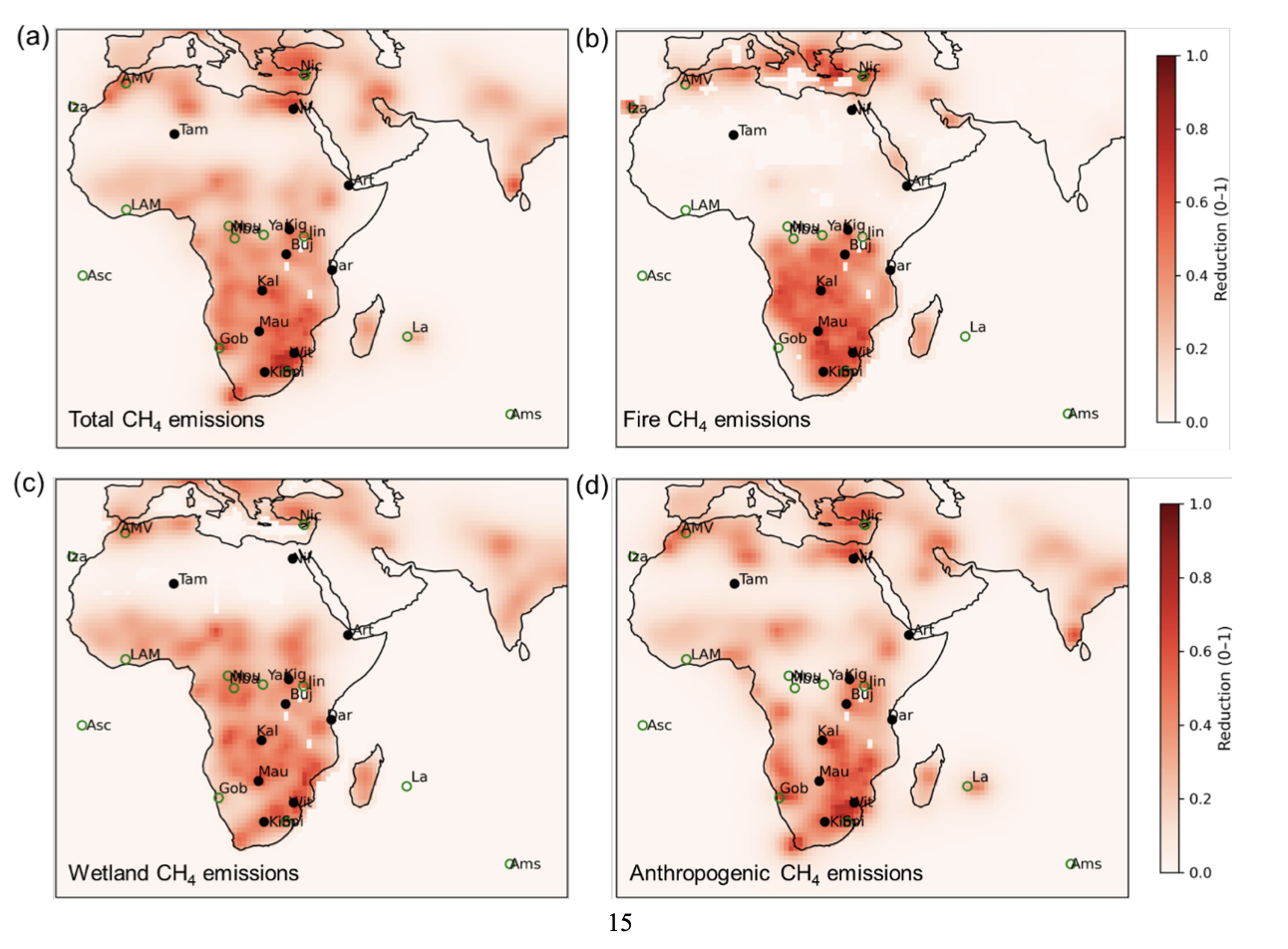


Fig S4. Theoretical uncertainty reduction for total emissions (a) and other sources (b-d) from an optimal network of 10 ground based EM27 sites. Lack of data during cloudy days at potential sites is included in the theoretical calculation (from Li *et al.* [7])

# References

1. Ciais, P., Y. Zhu, Y. Cai, et al. “Why Methane Surged in the Atmosphere during the Early 2020s.” *Science* 391, no. 6785 (2026): eadx8262. https://doi.org/10.1126/science.adx8262.

2. Kendall, M. G. *Rank Correlation Methods*. Griffin, 1948.

3. Mann, Henry B. “Nonparametric Tests Against Trend.” *Econometrica* 13, no. 3 (1945): 245–59. https://doi.org/10.2307/1907187.

4. Zhang, Zhen, Benjamin Poulter, Andrew F. Feldman, et al. “Recent Intensification of Wetland Methane Feedback.” *Nature Climate Change* 13, no. 5 (2023): 430–33. https://doi.org/10.1038/s41558-023-01629-0.

5. Yuan, K. “Africa Dominates the Long-Term Increase in Global Wetland CH_4_ Emissions Due to Warmer and Wetter Conditions.” *Nature*, 2026, In revision.

6. Zhu, Qing, Kunxiaojia Yuan, Fa Li, et al. “Critical Needs to Close Monitoring Gaps in Pan-Tropical Wetland CH4 Emissions.” *Environmental Research Letters* 19, no. 11 (2024): 114046. https://doi.org/10.1088/1748-9326/ad8019.

7. Li, Hui, Philippe Ciais, Frederic Chevallier, et al. “Strategic Design of Methane Observation Networks to Improve Emission Estimates: A Case Study in Africa.” *EGUsphere*, April 30, 2026, 1–23. https://doi.org/10.5194/egusphere-2026-1832.
